# The Gut Microbiota of Healthy and *Flavobacterium psychrophilum*-Infected Rainbow Trout Fry Is Shaped by Antibiotics and Phage Therapies

**DOI:** 10.3389/fmicb.2022.771296

**Published:** 2022-05-10

**Authors:** Valentina Laura Donati, Lone Madsen, Mathias Middelboe, Mikael Lenz Strube, Inger Dalsgaard

**Affiliations:** ^1^Unit for Fish and Shellfish Diseases, National Institute of Aquatic Resources, Technical University of Denmark, Kongens Lyngby, Denmark; ^2^Marine Biological Section, University of Copenhagen, Helsingør, Denmark; ^3^Department of Biotechnology and Biomedicine, Technical University of Denmark, Kongens Lyngby, Denmark

**Keywords:** *Flavobacterium psychrophilum*, RTFS, gut microbiota, microbiome, 16S rRNA gene, phage therapy, antibiotic, florfenicol

## Abstract

In the aquaculture sector, there is an increased interest in developing environmentally friendly alternatives to antibiotics in the treatment and prevention of bacterial infections. This requires an understanding of the effects of different treatments on the fish microbiota as a measure for improving the fish health status. In this study, we focused on the freshwater pathogen *Flavobacterium psychrophilum* and investigated the effects of antibiotics (florfenicol) and phage therapies on the gut microbiota of healthy and infected rainbow trout fry (1–2 g). Florfenicol-coated feed was administered for 10 days, starting two days after the infection procedure. A two-component mix of phage targeting *F. psychrophilum* (FpV4 and FPSV-D22) was continuously delivered by feed with a prophylactic period of 12 days. Samples of the distal intestine were collected over time (day -1 and 1, 8, and 33 days post-infection) and analyzed by community analysis targeting the 16S rRNA gene (V3–V4 region). Results showed the dysbiosis effect caused both by the infection and by florfenicol administration. Shifts in the overall composition were detected by β-diversity analysis, and changes in specific populations were observed during taxonomic mapping. Measures of α-diversity were only affected in infected fish (large variation observed 1 and 8 dpi). These community alterations disappeared again when fish recovered from the infection and the antibiotic treatment was terminated (33 dpi). Interestingly, phage addition altered the microbiota of the fish independently of the presence of their target bacterium. The overall gut bacterial community in fish fed phage-treated feed was different from the controls at each time point as revealed by β-diversity analysis. However, it was not possible to identify specific bacterial populations responsible for these changes except for an increase of lactic acid bacteria 33 dpi. Overall, the results indicate that the administered phages might affect the complex network of phage-bacteria interactions in the fish gut. Nevertheless, we did not observe negative effects on fish health or growth, and further studies should be directed in understanding if these changes are beneficial or not for the fish health with an additional focus on the host immune response.

## Introduction

The growth in production of the aquaculture sector (World Bank., 2013; FAO, 2018) has led to an increased interest in environmentally friendly alternatives to treat or prevent bacterial infections (reviewed by Culot et al., 2019; Kowalska et al., 2020). Moreover, understanding the effects of different treatments on the microbiota of the fish, as a measure for improving the fish health status, has a renewed interest (Perry et al., 2020). The gut microbiota, defined as the set of commensal, pathogenic, and symbiotic microorganisms (bacteria, archaea, viruses, bacteriophages, and fungi) inhabiting the gut, plays, indeed, a very important role in the development, growth, and health of the host (metabolic and digestive processes, the energy homeostasis by feeding regulation and the immune response) (Ingerslev et al., 2014a,b; Butt and Volkoff, 2019). It represents the most investigated microbiota in teleost compared to skin and gills, especially about diet (Perry et al., 2020). Several abiotic and biotic factors can influence the gut microbiota as the administration of probiotics (Gonçalves and Gallardo-Escárate, 2017), the presence of disease/infection (Ingerslev et al., 2014a; Nie et al., 2017), and antibiotic therapies (He et al., 2017; Gupta et al., 2019; Kim et al., 2019; reviewed by Butt and Volkoff, 2019).

*Flavobacterium psychrophilum* (Borg, 1948; Bernardet et al., 1996) is a freshwater pathogen causing important economic losses worldwide, as well as the etiological agent of Rainbow Trout Fry Syndrome (RTFS) and Bacterial Cold Water Disease (BCWD) (Borg, 1960; Lorenzen et al., 1991; reviewed by Nematollahi et al., 2003; Wahli and Madsen, 2018). To overcome the limitations and concerns concerning the standardly used antibiotic therapies, the utilization of bacteriophages (the so-called “phage-therapy”) is receiving increased attention as an alternative method, but also as prophylaxis, for the treatment and prevention of this bacterial infection (Stenholm et al., 2008; Madsen et al., 2013; Christiansen et al., 2014; Donati et al., 2021b).

Focusing on *F. psychrophilum* infections, we wanted to evaluate the effects of orally administered bacteriophages and florfenicol (antibiotic in use in Denmark for the treatment of RTFS) on the gut bacterial community of healthy and infected rainbow trout fry. In order to minimize the number of fish utilized in the experiments, this study was performed in combination with Experiment A in Donati et al. (2021b), where *F. psychrophilum* phages were applied on feed pellets by spraying or by irreversible immobilization (Mattey, 2016, 2018) and delivered to rainbow trout fry to evaluate the phage diffusion in fish internal organs, and the effects on fish survival during *F. psychrophilum* infections. In addition to the phage administration, groups of healthy and infected fish fed with antibiotics were included in the experimental trials and samples of the distal intestine were collected for all groups. A community analysis targeting the 16S rRNA gene (V3–V4 region) of the DNA extracted from intestine samples was performed with the aims of: (a) characterizing the microbiome, defined as the collection of genomes of the bacterial communities inhabiting the gut, of healthy rainbow trout fry fed with commercial feed and with phage-treated feed (after 11 days of prophylaxis); (b) evaluating the effects of florfenicol on the microbiome of healthy and infected fish during the treatment; and (c) observing the composition of the gut microbiome in fish that recovered from the infection under the different feed type regimes (commercial, phage-treated and antibiotic-coated feed). The work demonstrates the dysbiosis of the gut microbiome of rainbow trout fry during *F. psychrophilum* infection and florfenicol administration, but also suggests that this condition is lost once the fish have recovered from the infection and the antibiotic therapy is terminated. Further, this study demonstrates that orally administered bacteriophages can shape the gut microbial communities independently of the presence of their target pathogen.

## Materials and Methods

### Bacterial Strain

A well-characterized Danish strain of *Flavobacterium psychrophilum* was selected for the experiment (*F. psychrophilum* 950106-1/1; serotype Fd; virulent) (Madsen and Dalsgaard, 1999, 2000; Dalsgaard and Madsen, 2000; Sundell et al., 2019). The Swedish isolate *F. psychrophilum* FPS-S6 (serotype Th, virulent), was used for the propagation of the phage FPSV-D22, as this isolate is the most efficient host for the production of high titers of this specific phage (Sundell et al., 2019). The bacteria were stored in tryptone yeast extract salts medium (TYES) (Holt et al., 1993) and glycerol (15–20%) at –80°C. For bacteriophage titration, *F. psychrophilum* 950106-1/1 was prepared as described by Donati et al. (2021b). A 48–72 h broth culture of *F. psychrophilum* 950106-1/1 was prepared in 5 ml of TYES broth (referred to as TYES-B) from a –80°C stock (incubation at 15°C at 100 rpm) and streaked on TYES agar (referred as TYES-A: TYES-B containing 1.1% agar). Following an incubation period of 3–4 days at 15°C, single colonies were inoculated in TYES-B (5 ml) for 48 h and, then, used for the phage quantification assay. For the experimental fish challenge, *F. psychrophilum* 950106-1/1 was prepared and the infection challenge was performed as described by Madsen and Dalsgaard (1999). Briefly, a 48-h culture was diluted in TYES-B and 50 μl of the selected dilution were injected in the peritoneal cavity (intraperitoneal injection, IP) of the fish (1.98 ± 0.65 g, *n* = 20) for a final dose of 1*10^4^ colony-forming unit (CFU) fish^–1^ [the low infection dose was selected as we wanted to increase the multiplicity of infection (MOI) to allow the phages to control the bacterial infection]. Control fish were injected with 50 μl of sterile TYES-B. The CFU count of the injected culture was performed before and after infection, in duplicates. This method of infection was selected as this is considered the most reproducible in the case of experimental *F. psychrophilum* infection challenge in rainbow trout fry (Madsen and Dalsgaard, 1999; Donati et al., 2021b).

### Bacteriophages

Two Danish lytic bacteriophages infecting *F. psychrophilum* were selected for the experiment: FpV4 (*Podoviridae* family, 90 kb genome, isolated in 2005) (Stenholm et al., 2008; Castillo and Middelboe, 2016) and FPSV-D22 (*Siphoviridae* family, 42.7 kb genome, isolated in 2017) (Sundell et al., 2019; Castillo et al., 2021; Donati et al., 2021b). Both phages were characterized to have a broad host range among *F. psychrophilum* strains, stability of up to 2 months in filtered fresh water (in the dark at 15°C), high production efficiency, and absence of unwanted genes (e.g., antibiotic resistance) (Stenholm et al., 2008; Castillo et al., 2014, 2021; unpublished data). An overview of the characteristics of these two lytic phages can be found in the Supplementary Materials of Donati et al. (2021b). Purified highly concentrated solutions of FpV4 and FPSV-D22 were stored at –80°C with SM buffer (8 mM MgSO_4_, 99 mM NaCl, 50 mM Tris–Cl (pH 7.5), 0.01% gelatin) and glycerol (15%) (Stenholm et al., 2008; Sundell et al., 2019). The two phages were used in a series of parallel experiments with the overall aim of obtaining a high combinatory effect on pathogen control, based on their lytic properties (e.g., Donati et al., 2021a). So, to maintain a systematic approach to our phage control studies, we have used the same combination of well-characterized phages in all the studies.

The quantification of plaque-forming units (PFU) in phage solutions was performed by the spot test method (Stenholm et al., 2008; Clokie and Kropinski(eds), 2009). Briefly, a bacterial lawn was prepared by mixing 300 μl of a 48-h old *F. psychrophilum* broth culture with 4 ml of TYES soft agar (0.4% agar) and the mixture was poured onto a TYES-A plate. Five microliters of undiluted samples were spotted on the bacterial lawn in triplicates and incubated at 15°C for 3–4 days. Spots with single plaques (from one to 30) were counted. In the case of confluent or semi-confluent clearing areas, 10-fold dilutions were performed (180 μl of SM buffer and 20 μl of the sample) in triplicates and spotted on a bacterial lawn. Plates were incubated at 15°C for 3–4 days, single plaques were counted, and the phage titer was calculated. For quantification of phages on feed pellets (Christiansen et al., 2014; Donati et al., 2021b), 0.1 g of feed (*n* = 3) and 2 ml of SM buffer were mixed in 2 ml sterile microtubes (SARSTEDT AG & Co. KG, Nümbrecht, Germany) for each feed type (phage treated pellets and control feed), a sterile 5-mm steel bead (Qiagen, Hilden, Germany) was added and samples were homogenized for 1 min at 20 Hrz with a Qiagen TissueLyser II (Qiagen, Hilden, Germany). Homogenized samples were stored for 1 h at 5°C and then, transferred to 15 ml sterile Falcon tubes with 3 ml of sterile SM buffer and vortexed. The PFU quantification per gram of feed was performed by the spot test method as described above.

### Feed Types

Four feed types were selected for our experiment: control feed (named C); florfenicol-coated feed (named An, short for “Antibiotic”); phage-immobilized feed (named PI), and phage-sprayed feed (named PS). Feed pellets (0.8 mm, Inicio Plus, BioMar A/S, Brande, Denmark) were used as control feed and for applying either the antibiotic or the selected phages. The commercial feed type was selected as the starter feed for the rainbow trout fry. The composition of the commercial feed pellets, Inicio Plus, used for the groups C, An, PI, and PS is the following (BioMar A/S, 2020): 60.3% protein, 33% lipid, 6.7% starch plus Bactocell^®^, a probiotic lactic acid bacterium (*Pediococcus acidilactici*), and B-WYSE™ (BioMar Whole Yeast Synergistic Extracts), a yeast-based additive, developed by BioMar’s partner Lallemand Animal Nutrition (Canada).

The preparation of phage-treated feed pellets was done as follows [for more details, see description of experiment A in Donati et al. (2021b)]. Briefly, high titer solutions of phages FpV4 and FPSV-D22 were prepared from crude lysates following infection of *F. psychrophilum* strains FPS-S6 (for FPSV-D22 propagation) and 950106-1/1 (for FpV4 propagation), filtered through a 0.2 μm pore size sterile filter, concentrated by adding poly-ethylene glycol 8000 (PEG-8000) and sodium chloride (final concentration 10% w/v and 1 M, respectively), centrifuged (10,000 × *g*, 30 min, 4°C) after 24 h incubation at 4°C and re-suspended in sterile SM buffer (Castillo et al., 2019; Donati et al., 2021b). The purified high titer solutions of FpV4 (1.2*10^9^ PFU ml^–1^) and FPSV-D22 (4.9*10^9^ PFU ml^–1^) were mixed 1:1 [total phage concentration of 3.3*10^9^ ± 6.1*10^8^ PFU ml^–1^ (mean ± SD, *n* = 3)]. The two-component phage mix was applied on control feed pellets from the same batch by irreversible immobilization using the corona discharge technology by Fixed Phage Ltd (20 ml per 100 g; phage-immobilized feed, PI) (Mattey, 2016, 2018) or by spraying (30 ml per 100 g; phage-sprayed feed, PS) (Christiansen et al., 2014; Donati et al., 2021b). Phage-treated feed pellets were stored at 5°C in the dark. The measured final concentration of phages on feed pellets (performed as described in the previous paragraph) was 8.3*10^7^ ± 2.5*10^7^ PFU g^–1^ of PI (*n* = 3) and 1.6*10^8^ ± 4.8*10^7^ PFU g^–1^ of PS (*n* = 3).

Florfenicol-coated feed (An) was purchased and stored at 5°C in the dark. For this feed type, florfenicol (Aquaflor^®^, Intervet Inc., a subsidiary of Merck & Co. Inc., Kenilworth, NJ, United States) was applied on Inicio Plus (BioMar A/S, Brande, Denmark) feed pellets for a final concentration of 0.2% (veterinarian Thomas Clausen personal communication). The recommended administration for rainbow trout fry was 2% of fish weight per day for 10 consecutive days.

### Experimental Setup

Rainbow trout eyed eggs were purchased at a Danish commercial fish farm, iodophor disinfected, hatched, and the fish were grown at the Unit for Fish and Shellfish Diseases (DTU Aqua, Kgs. Lyngby, Denmark). Initially raised in a recirculation system, fish were then transferred to a dedicated experimental area (flow-through system, no recirculation of water) used for experimental work when the desired size/weight was reached. Fish were divided randomly into 8-L tanks, each with a separate inlet/outlet for water (13°C) and air supply.

**FIGURE 1:**
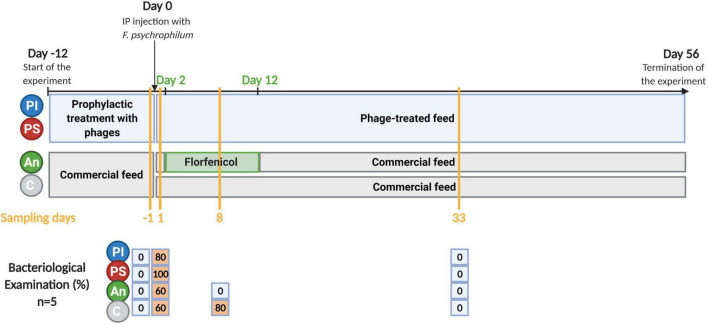
Timeline of the experiment. C, control feed group; An, antibiotic feed group; PI, phage-immobilized feed group; PS, phage-sprayed feed group. The results of the bacteriological examination performed on sampled fish are presented (% of fish positive to re-isolation of *F. psychrophilum*). Created with BioRender.com (the figure was exported under a paid subscription).

Rainbow trout fry (1–2 g) were divided into 16 × 8 L-aquaria (∼50 fish/aquarium) and the four feed groups (C, An, PI, and PS) with four aquaria per group. All groups were fed at 2% of fish weight per day. The PI and PS were administered continuously for 12 days before the bacterial challenge. The florfenicol-coated feed was administered to the fish for 10 days starting 2 days after bacterial exposure. When not fed with antibiotic-coated feed, fish in this group (An) were fed with control feed (Figure 1). Fish in three of the four aquaria per treatment group were challenged with *F. psychrophilum* 950106-1/1 by injection as described above [indicated by “feed type/bacterial strain (Fp)”]. As controls for the infection, fish in one aquarium per treatment group were injected with sterile TYES-B. Prior to IP injection, fish were anesthetized with 3-aminobenzoic acid ethyl ester (MS-222, Sigma catalog number A-5040). Survival of fish was followed for each group in two of the infected aquaria. Fish sampling was performed in the remaining two (one infected with the bacterium and one non-infected) during the experiment. Dead and moribund fish were collected, and moribund fish were euthanized with an overdose of MS-222. The length and weight of each fish were recorded, and bacteriological examination was performed as described for sampled fish (see below).

The results of the fish survival (C, PI, and PS incubations) and the re-isolation/quantification of bacteria and phages in the intestine and the internal organs of the fish fed with PI and PS, are presented in Donati et al. (2021b) (Experiment A). The final percentage survival for fish fed with PS, PI, and C was 75.6, 80.1, and 76.8%, respectively, with no significant difference among feed groups. No mortality was recorded in fish fed antibiotic feed.

The use of fish in this study complied with Danish and EU legislation (Directive 2010/63/EU) on animal experimentation, and it was approved by the Animal Experiments Inspectorate of Denmark (Dyreforsøgstilsynet, permission number 2013-15-2934-00976 until 7/10-19, and 2019-15-0201-00159 from 8/10-19).

### Sampling for Microbiome Analysis

One day before the bacterial exposure, five fish were sampled from each feed-type group from the sampling aquaria not supposed to be infected with *F. psychrophilum* (C, An, PI, and PS). Following the bacterial challenge, five fish from each sampling aquaria (C, An, PI, PS, C/Fp, An/Fp, PI/Fp, and PS/Fp) were sampled at 1 and 33 days post-infection (dpi) (Figure 1). Eight dpi, five fish from the control, and the antibiotic groups with and without the infection were also sampled (C, An, C/Fp, and An/Fp). Fish were euthanized with an overdose of MS-222 and, their weights and lengths were measured. Bacteriological examination of the spleen, kidney, and brain was performed by streaking samples of organs on TYES agar plates. Plates were incubated at 15°C for 3–5 days until a maximum of 4 weeks and the growth of yellow *F. psychrophilum* colonies was recorded. Randomly chosen colonies were then analyzed by MALDI-TOF MS (Bruker Daltonic GmbH, Bremen, Germany) to confirm that *F. psychrophilum* was the re-isolated bacteria (Donati et al., 2021b). For microbiome analysis, the distal part of the intestine was aseptically removed together with the fecal content, if present, and placed in a sterile 1.5 ml microtube (SARSTEDT AG & Co. KG, Nümbrecht, Germany). Due to the small size of the fish, it was chosen to collect the distal intestine with the content to avoid any unintentional manipulation/modification of the microbial community. Intestine samples were stored at –20°C (Ingerslev et al., 2014a,b). Fish were not fed 24 h before sampling to diminish the possibility of the presence of gut content in the fish.

### DNA Extraction

DNA extraction was performed using the Maxwell LEV Blood DNA Purification Kit (Promega Corporation, Madison, WI, United States) (Ingerslev et al., 2014a,b; Strube et al., 2018). After thawing, samples were transferred to sterile 2 ml Eppendorf tubes containing a sterile 5 mm steel bead (Qiagen, Hilden, Germany) and incubated with 100 μl of lysozyme mixture [25 mg/ml lysozyme, 1.2% Triton X, 2 mM EDTA, and 20 mM Tris-HCl (pH 8)] for 1 h at 37°C. Subsequently, 350 μl of lysis buffer were added and each sample was homogenized by using a Qiagen TissueLyser II (2 min at 20 Hrz; Qiagen, Hilden, Germany). Samples were incubated overnight at 56°C with 20 μl Proteinase K (20 mg/ml). A Maxwell 16 Research Instrument System (Promega Corporation, Madison, WI, United States) was used following the manufacturer’s instructions. The concentration and quality of the extracted DNA were measured by a NanoDrop One Microvolume UV-Vis Spectrophotometer (Thermo Fisher Scientific, Wilmington, DE, United States) and with a Qubit 1X dsDNA HS (High Sensitivity) assay kit (catalog number Q33231, Invitrogen by Thermo Fisher Scientific, Life Technologies Corporation, Eugene, OR, United States) in the Invitrogen Qubit™ 4 Fluorometer (Invitrogen by Thermo Fisher Scientific, Life Technologies Holdings Pte Ltd, Singapore). A negative control (empty 2 ml vial in which the bead and the various solutions for the DNA extraction and purification were added during the procedure) was included in the DNA extraction and purification protocol.

### Library Preparation

Extracted DNA was sent to DNASense (Aalborg, Denmark) for 16S DNA library preparation, sequencing, and bioinformatics, which were performed as follows. Bacterial 16S rRNA gene region V3-4 sequencing libraries were prepared by a custom protocol based on an Illumina protocol (Illumina, 2015). Approximately 15 ng of extracted DNA was used as template for PCR amplification of the Bacteria 16S rRNA gene region V3-4 amplicons. Each PCR reaction (25 μL) contained 12.5 μL PCRBIO Ultra mix (PCR Biosystems, Wayne, PA, United States) and 400 nM of each forward and reverse tailed primer mix. A PCR was conducted with the following program: initial denaturation at 95°C for 2 min, 30 cycles of amplification (95°C for 15 s, 55°C for 15 s, and 72°C for 50 s), and a final elongation at 72°C for 5 min. Duplicate PCR reactions were performed for each sample and the duplicates were pooled after PCR. The forward and reverse tailed primers were designed according to Illumina (2015) and contain primers targeting the Bacteria 16S rRNA gene region V3-4: [341F] CCTACGGGNGGCWGCAG and [805R] GACTACHVGGGTATCTAATCC (Herlemann et al., 2011). The primer tails enable the attachment of Illumina Nextera adaptors that are necessary for sequencing in a subsequent PCR. The resulting amplicon libraries were purified using the standard protocol for Agencourt Ampure XP Beads (Beckman Coulter, Indianapolis, IN, United States), with a bead to sample ratio of 4:5. The DNA was eluted in 25 μL on nuclease-free water (Qiagen, Hilden, Germany). The DNA concentration was measured using the Qubit dsDNA HS Assay kit (Invitrogen by Thermo Fisher Scientific, Life Technologies Corporation, Eugene, OR, United States). Gel electrophoresis using Tapestation 2200 and D1000/High-sensitivity D1000 screentapes (Agilent, Santa Clara, CA, United States) was used to validate the product size and purity of a subset of sequencing libraries. Sequencing libraries were prepared from the purified amplicon libraries using a second PCR. Each PCR reaction (25 μL) contained PCRBIO HiFi buffer (1x), PCRBIO HiFi Polymerase (1 U/reaction) (PCR Biosystems, London, United Kingdom), adaptor mix (400 nM of each forward and reverse) and up to 10 ng of amplicon library template. A PCR was conducted with the following program: Initial denaturation at 95°C for 2 min, 8 cycles of amplification (95°C for 20 s, 55°C for 30 s, and 72°C for 60 s) and final elongation at 72°C for 5 min. The resulting sequencing libraries were purified using the standard protocol for Agencourt Ampure XP Beads (Beckman Coulter, Indianapolis, Indiana, United States), with a bead to sample ratio of 4:5. The DNA was eluted in 25 μL of nuclease-free water (Qiagen, Hilden, Germany). The DNA concentration was measured using the Qubit dsDNA HS Assay kit (Invitrogen by Thermo Fisher Scientific, Life Technologies Corporation, Eugene, OR, United States). Gel electrophoresis using Tapestation 2200 and D1000/High sensitivity D1000 screentapes (Agilent, Santa Clara, CA, United States) was used to validate the product size and purity of a subset of sequencing libraries.

### DNA Sequencing and Bioinformatics Processing (DNASense)

The purified sequencing libraries were pooled in equimolar concentrations and diluted to 2 nM. The samples were paired- end sequenced (2 × 300 bp) on a MiSeq (Illumina, United States) using a MiSeq Reagent kit v3 (Illumina, United States), following the standard guidelines for preparing and loading samples on the MiSeq. More than 10% of PhiX control library was spiked in.

Forward and reverse reads were trimmed for quality using Trimmomatic v. 0.32 (Bolger et al., 2014) with the settings SLIDINGWINDOW: 5:3 and MINLEN: 275. The trimmed forward and reverse reads were merged using FLASH v. 1.2.7 (Magoč and Salzberg, 2011), with the settings -m 10 -M 250. The trimmed reads were dereplicated and formatted for use in the UPARSE workflow (Edgar, 2013). The dereplicated reads were clustered, using the usearch v. 7.0.1090 -cluster_otus command with default settings. The operational taxonomic unit (OTU) abundances were estimated using the usearch v. 7.0.1090 -usearch_global command with -id 0.97 -maxaccepts 0 -maxrejects 0. Taxonomy was assigned using the ribosomal database project (RDP) classifier (Wang et al., 2007), as implemented in the parallel_assign_taxonomy_rdp.py script in QIIME (Caporaso et al., 2010), using –confidence 0.8 and the SILVA database, release 132 (Quast et al., 2013). The results were analyzed in R v. 4.0.2 (R Core Team, 2017) through the Rstudio integrated development environment (IDE) using the ampvis package v.2.6.5 (Albertsen et al., 2015).

### Statistics

The analyses were performed with GraphPad Prism version 8.4.0 for Windows (GraphPad Software, San Diego, CA, United States, www.graphpad.com). The OTUs relative abundances, the Shannon diversity index, the Chao1 richness index, and fish size were analyzed to assess if there was any statistically significant difference among the groups. First, normality was evaluated with the Shapiro–Wilk test. Comparisons were then performed with ANOVA or Krustal–Wallis in case of non-normal data distribution. The *P*-values (*P*) below 0.05 were considered significant. The *P*-values for multiple comparisons were adjusted for Dunnet (normal distribution) or Dunn’s (non-normal distribution) corrections. Shannon diversity index values are based on 10,000 reads per sample. For comparison of the abundance of specific bacteria of interest between one group at two different time points (e.g., the genus *Rhodococcus* in C), the Mann–Whitney test was used (*P* < 0.05 is considered significant).

The Principal Coordinates Analysis (PCoA) based on the Bray-Curtis distance measure (Bray and Curtis, 1957) was used to assess β-diversity and the group-wise differences were tested with permutational multivariate analysis of variance (PERMANOVA) using the adonis function from the vegan package (Oksanen et al., 2020).

## Results

### Library Preparation and Sequencing (DNASense)

Library preparation for bacterial sequencing (V3–4) was successful in all samples except for one, which yielded 30 reads after quality control (QC) and bioinformatic processing (and so it was excluded). Seven low-input samples yielded only ∼4,600–8,000 reads, but the general sequencing outcome was ∼10,000 or more reads. One sample produced > 400,000 reads, which was attributable to the overloading of that sample when pooling sequencing libraries (Supplementary Table 1). The negative controls implemented in the project revealed minor reagent contamination from 2 specific OTUs, namely, OTU 1 belonging to the genus *Pseudomonas* and OTU 4 belonging to the genus *Sphingobacterium*. Noteworthy, the fraction of total OTU counts attributed to the two contaminating OTUs showed an inverse relationship with PCR amplicon yield, i.e., the two OTUs were only an issue in samples with low bacterial DNA input material. Comparing laboratory journals across projects processed in parallel identified the likely source as batch PCR primers provided by the supplier. However, the two OTUs had a negligible effect on project outcome, and the OTUs were excluded from downstream analyses.

### Microbial Gut Community of Rainbow Trout Fry and Effects of Phage Prophylaxis (Day -1)

At first, the gut microbiome of rainbow trout fry was studied 1 day prior to infection (fish negative to *F. psychrophilum*). At this time point, C and An were fed with non-treated feed, while PI and PS had received phages for 11 days (Figure 1).

**FIGURE 2:**
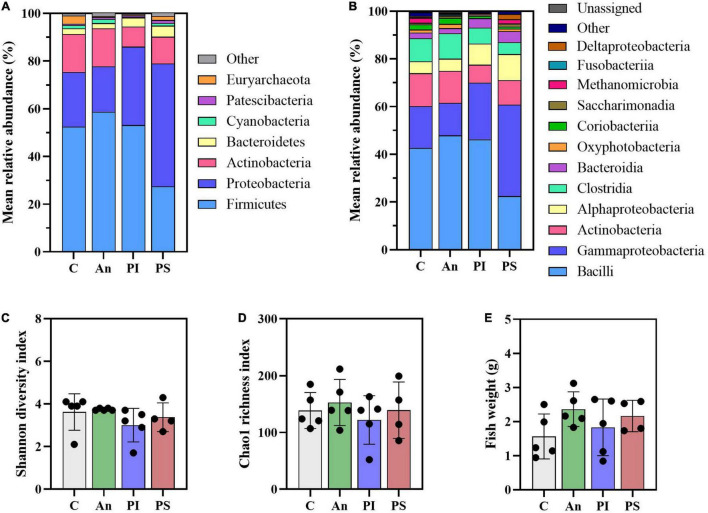
Microbial community (day -1). Characterization of mean relative abundance at phylum **(A)** and class **(B)** level, Shannon diversity index **(C)**, Chao1 richness index **(D),** and fish weight **(E)**. Values represent the mean of five samples except for group fed with phage-sprayed feed (*n* = 4). In **(C–E)**, error bars represent the standard deviation. Shannon diversity index values are based on 10,000 reads per sample. C, control feed group; An, antibiotic feed group; PI, phage-immobilized feed group; PS, phage-sprayed feed group.

The taxonomic mapping at phylum and class level (Figures 2A,B) did not reveal any statistically significant difference among the top-5 and top-7 most abundant phyla and classes, respectively (Supplementary Tables 2, 3). The dominant phyla were Firmicutes (C: 52.4 ± 11.4%), Proteobacteria (C: 23.0 ± 16.0%) and Actinobacteria (C: 16.0 ± 12.0%), followed by Bacteroidetes (C: 2.4 ± 2.6%) and Cyanobacteria (C: 1.3 ± 1.4%). The top-7 most abundant classes were Bacilli (C: 42.6 ± 9.6%), Gammaproteobacteria (C: 17.6 ± 13.5%), Actinobacteria (C: 13.8 ± 12.1%), Alphaproteobacteria (C: 4.9 ± 2.8%), Clostrida (C: 9.7 ± 5.6%), Bacteroida (C: 2.4 ± 2.6%) and Oxyphotobacteria (C: 1.3 ± 1.4%). Furthermore, no statistically significant differences in the Shannon diversity index (C: 3.6 ± 0.9), the Chao1 richness index (C: 138.6 ± 31.6) and the fish size (C: 1.7 ± 0.7 g) were observed among the feed groups (Figures 2C–E).

**FIGURE 3:**
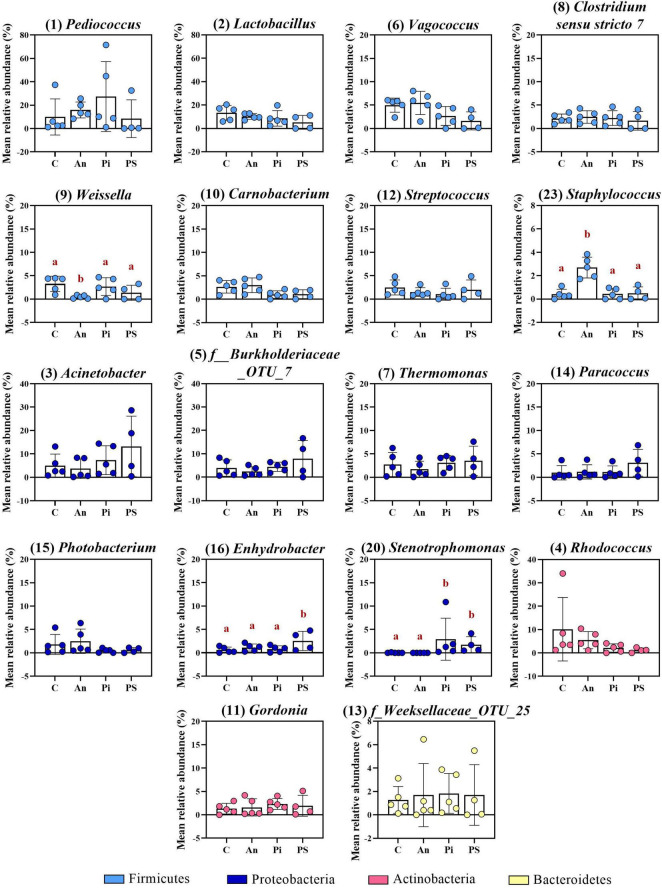
Top-16 most abundant, plus two additional genera, selected among the top-30 (day -1). The selection of the additional genera was based on abundances presenting statistically significant differences to C (all top-30 genera were tested). The number in parenthesis states the position of the genus in the ranking. Values represent the mean and SD of five samples except for group fed with phage-sprayed feed (*n* = 4). Differences are tested by ANOVA or Kruskal–Wallis (*P*-values are adjusted for multiple comparison). When significant, differences are presented with different red letters. C, control feed group; An, antibiotic feed group; PI, phage-immobilized feed group; PS, phage-sprayed feed group. See Supplementary Table 4 for more information on the top-30 most abundant genera at day -1.

The top-30 most abundant genera are presented in Figure 3 and Supplementary Table 4 and, among them, *Lactobacillus* (C: 13.4 ± 6.4%), *Rhodococcus* (C: 10.2 ± 13.6%), *Pediococcus* (C: 9.9 ± 15.5%), *Acinetobacter* (C: 5.0 ± 5.0%), *Vagococcus* (C: 5.0 ± 1.5%) and *Thermomonas* (C: 2.7 ± 2.6%) were observed as the most copious. Even though fish in C and An received the same feed type at this time point and no significant differences were observed at phylum or class level, dissimilarities were observed: a higher abundance of *Staphylococcus* (Phylum: Firmicutes; Class Bacilli; Order Bacillales) (2.7 ± 0.9%; adjusted *P* < 0.0001) and a lower abundance of *Weissella* (Phylum: Firmicutes; Class Bacilli; Order Lactobacillales) (0.5 ± 0.3%; adjusted *P* = 0.024) were observed in An relative to the control (C). Additional differences concerning fish fed with phage-treated feed pellets compared to C were revealed by the taxonomic mapping at genus level as significantly higher abundances of *Enhydrobacter* (Phylum Proteobacteria; Class Alphaproteobacteria; Order Rhodospirillales) and *Stenotrophomonas* (Phylum Proteobacteria; Class Gammaproteobacteria; Order Xanthomonadales) were observed for PS (2.5 ± 2.1%; adjusted *P* = 0.049) and, PS and PI (in PS: 1.7 ± 1.7%, adjusted *P* = 0.048; in PI: 2.9 ± 4.5%, adjusted *P* = 0.046), respectively.

**FIGURE 4:**
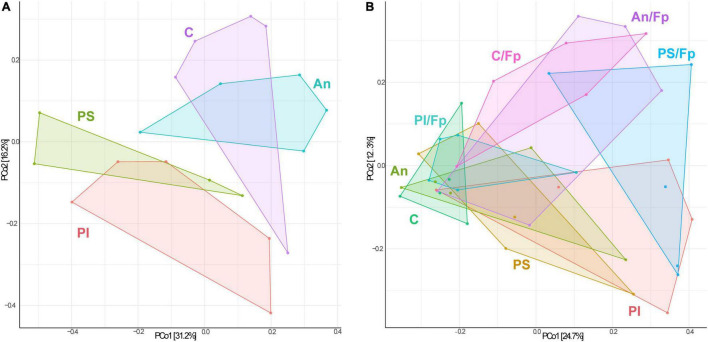
Principal Coordinates Analysis (PCoA) 1 day before (**A**, 19 samples and 423 OTUs) and 1 day after (**B**, 40 samples and 478 OTUs) the infection [The OTUs were transformed to relative abundance]. Prior to the analysis, OTU’s that are not present in more than 0.1% relative abundance in any sample have been removed. No initial data transformation has been applied. The relative contribution (eigenvalue) of each axis to the total inertia in the data is indicated in percent at the axis titles. C, control feed group; An, antibiotic feed group; PI, phage-immobilized feed group; PS, phage-sprayed feed group; C/Fp, control feed group + *F. psychrophilum*; An/Fp, antibiotic feed group + *F. psychrophilum*; PI/Fp, phage-immobilized feed group + *F. psychrophilum*; PS/Fp, phage-sprayed feed group + *F. psychrophilum*.

The identification of similarities/dissimilarities among the microbial communities of the fish gut (β-diversity) was performed using PCoA (Figure 4A). The clusters formed by fish fed phage-treated feed (PS and PI) were separated from the clusters of C and An, suggesting an effect of the phage treatments on the microbial community (*P* = 0.002, PERMANOVA). No statistically significant difference was detected between the microbiome of C and An (*P* = 0.256, PERMANOVA) and between PI and PS (*P* = 0.326, PERMANOVA).

### Effects of Bacterial Infection and Infection Procedure (Microbial Gut Community 1 dpi)

One day post infection, fish were sampled to evaluate the effect of the infection and at this time point, 60, 60, 80, and 100% of sampled fish in the groups C/Fp, An/Fp, PI/Fp, and PS/Fp, respectively, were positive to *F. psychrophilum* (Figure 1). Sampled fish in control aquaria without IP injection with *F. psychrophilum* (C, An, PI, and PS) were all negative to the bacterium. As for day -1, fish in C, An, C/Fp, and An/Fp were fed with non-treated feed.

The PCoA analysis revealed dissimilarities among the bacterial community between the feed groups (Figure 4B). Fish in An were characterized by a different community than C (*P* = 0.033, PERMANOVA). The clusters formed by PS and PI were divergent from the cluster of C and An (*P* = 0.028, PERMANOVA), also indicating a larger in-group variation among the microbial communities of PI and PS. No statistically significant difference was detected between the microbiome of PI and PS (*P* = 0.133, PERMANOVA). Furthermore, a diversification of the bacterial communities was observed when fish were exposed to *F. psychrophilum* (C/Fp, An/Fp, PI/Fp, and PS/Fp) compared to non-challenged fish (*P* = 0.016, PERMANOVA). The groups C and PS/Fp formed two independent clusters. While no statistically significant difference was detected between C/Fp and An/Fp (*P* = 0.091, PERMANOVA), this was not the case between PI/Fp and PS/Fp (*P* = 0.018, PERMANOVA).

**FIGURE 5:**
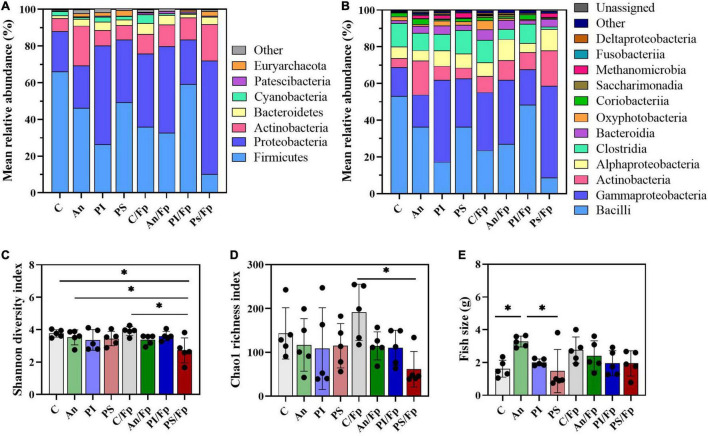
Microbial community at 1 dpi. Characterization of mean relative abundance at phylum **(A)** and class **(B)** level, Shannon diversity index **(C)**, Chao1 richness index **(D)**, and fish weight **(E)**. Values represent the mean of five samples. In **(C–E)**, error bars represent the standard deviation and statistically significant differences are indicated by * (adjusted *p*-value < 0.05). Shannon diversity index values are based on 10,000 reads per sample. C, control feed group; An, antibiotic feed group; PI, phage-immobilized feed group; PS, phage-sprayed feed group; C/Fp, control feed group + *F. psychrophilum*; An/Fp, antibiotic feed group + *F. psychrophilum*; PI/Fp, phage-immobilized feed group + *F. psychrophilum*; PS/Fp, phage-sprayed feed group + *F. psychrophilum*.

**FIGURE 6:**
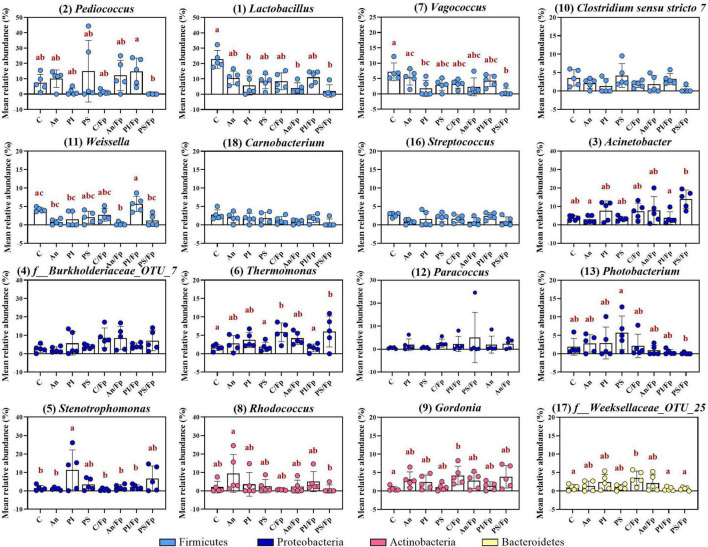
Top-13 most abundant, plus three additional genera, selected among the top-30 in fish fed with control, antibiotic, phage-immobilized and phage-sprayed feed with (C/Fp; An/Fp; PI/Fp; PS/Fp) and without (C; An; PI; PS) bacterial exposure (1 dpi). The selection of the additional genera was based on previously observed abundances (among top-13 at day -1). The number in parenthesis states the position of the genus in the ranking. Values represent the mean and SD of five samples. Differences are tested by ANOVA or Kruskal–Wallis (*P*-values are adjusted for multiple comparison). When significant, differences are presented with different red letters. See Supplementary Table 7 for more information on the top-30 most abundant genera at day 1.

Dissimilarities were also revealed by the taxonomic mapping [e.g., decline of Firmicutes reflected in a rise in Proteobacteria in PI, C/Fp, An/Fp, and PS/Fp (vs. C)] and alpha diversity values [i.e., lower Shannon diversity and Chao1 richness indexes in PS/Fp vs. C/Fp (adjusted *P* ≤ 0.01)] (Figures 5, 6 and Supplementary Tables 5–7). In contrast to the other time points, a variation about fish size was observed (Figure 5E), as fish in An (3.3 ± 0.3 g) were larger than fish in C (1.6 ± 0.5 g; adjusted *P* = 0.0389), likely contributing to the variation observed in the PCoA plot between these two groups.

Compared to the Firmicutes abundance in the control group C (66.0 ± 12.0%), a general drop was observed in PI (26.4 ± 24.5%), C/Fp (36.0 ± 15.3%), An/Fp (32.7 ± 18.0%) and, with the largest and significant decrease, in PS/Fp (10.2 ± 12.0%, adjusted *P* = 0.0020) (Figure 5A and Supplementary Table 5). These changes were reflected at class level in the abundances of Bacilli and Clostridia, which were significantly lower in PS/Fp than in C (Bacilli: PS/Fp: 8.7 ± 9.1%; C: 53.1 ± 11.7%; adjusted *P* = 0.0056) (Clostridia: PS/Fp: 1.3 ± 2.7%; C: 12.8 ± 3.8%; adjusted *P* = 0.0459) and than in PS in case of Clostridia (12.7 ± 5.3%; adjusted *P* = 0.0259) (Figure 5B and Supplementary Table 6). The general decrease in Bacilli abundance in PI, C/Fp, An/FP, and PS/Fp was observed at the genus level at different significant levels among the genera *Lactobacillus*, *Pediococcus*, *Vagococcus*, and *Weissella* (Order Lactobacillales) (Figure 6 and Supplementary Table 7). Specifically, the genus *Lactobacillus* was reduced in PI, C/Fp, An/FP, and PS/Fp compared to C (adjusted *P* ≤ 0.01). A significant decline in *Vagococcus* was detected in PI (1.8 ± 2.6%; adjusted *P* = 0.0264) and PS/Fp (0.6 ± 1.3%; adjusted *P* = 0.0025) compared to C (7.2 ± 2.9%). The genus *Pediococcus* was reduced in PI, C/Fp, and PS/Fp, and a significant difference was detected between PS/Fp (0.05 ± 0.1%) and PI/Fp (14.8 ± 8.7%) (adjusted *P* = 0.0116). Further, same as observed at day -1, the genus *Weissella* was characterized by lower abundances in An (0.8 ± 0.8%) and An/Fp (0.3 ± 0.4%) compared to C (4.1 ± 0.6%) (An/Fp vs. C: adjusted *P* = 0.0200). The differences observed among the genus *Staphylococcus* at day -1 between C and An were not observed at this time point as its relative abundance was around 1% in all groups, except in An/Fp where it was 4.4 ± 6.6% (adjusted *P* > 0.05).

The decline in Firmicutes seemed to favor a rise in Proteobacteria (Figures 5A,B). Compared to the Gammaproteobacteria abundance in the control group C (15.7 ± 5.1%), a general increase was detected in PI (44.7 ± 26.3%), C/Fp (31.7 ± 12.7%), An/Fp (34.9 ± 15.9%) and, with the largest increment, in PS/Fp (50.0 ± 17.4%, adjusted *P* = 0.0224) (Supplementary Table 6). These changes were reflected in the abundance of the genera *Stenotrophomonas*, *Thermomonas*, *Acinetobacter*, and *Photobacterium* (Figure 6 and Supplementary Table 7). The abundance of the genus *Thermomonas* (Order Xanthomonadales) was significantly increased in C/Fp (5.8 ± 2.5%; adjusted *P* = 0.0301) and PS/Fp (6.0 ± 4.2%; adjusted *P* = 0.0211) compared to C (1.7 ± 0.8%), as well as the genus *Stenotrophomonas* (Order Xanthomonadales) in PI (11.4 ± 10.8%), as at day -1. The genus *Acinetobacter* (Order Moraxellaceae) significantly expanded in PS/Fp (14.0 ± 4.5%) compared to An (3.0 ± 2.0%) and PI/Fp (3.7 ± 3.4%) (adjusted *P* < 0.05). Finally, fish in PS were characterized by a higher abundance of the genus *Photobacterium* (Order Vibrionales) compared to PS/Fp (PS: 5.7 ± 4.5%; PS/Fp: 0.1 ± 0.2%, adjusted *P* = 0.0048). Different from the observations at day -1, no significant differences were observed in the genus *Enhydrobacter* between PS and the other feed groups, even if a larger variation was observed for PS (0.9 ± 1.3%). Generally, a higher abundance of this genus was observed in fish exposed to *F. psychrophilum* (except PI/Fp) and a significant increment was detected in An/Fp (2.3 ± 1.5%) compared to PI (0.2 ± 0.3%; adjusted *P* = 0.0467).

Additional variations in the taxonomic mapping were revealed among the phyla Actinobacteria, Bacteroidetes, and Cyanobacteria (Figure 5A and Supplementary Table 5). A greater abundance of Actinobacteria was detected in An (An: 21.6 ± 12.1%; C: 7.0 ± 3.9%; adjusted *P* = 0.037). At the genus level (Figure 6 and Supplementary Table 7), variations were observed in the abundances of *Rhodococcus* and *Gordonia* (Class Actinobacteria; Order Actinomycetales). Compared to day -1, where the abundance of the genus *Rhodococcus* was 10.2 ± 13.6% in C, lower values were observed 1 dpi (C: 2.1 ± 3.2%) (Mann–Whitney test; *P* > 0.05). The highest quantity was detected in An (9.5 ± 10.3%) and the lowest in C/Fp (0.5 ± 0.2%) and PS/Fp (1.0 ± 2.2%). A rise in *Gordonia* was recorded in C/Fp (4.2 ± 2.5%) compared to C (0.6 ± 0.6%) (adjusted *P* < 0.05). Even if no significant differences in the phylum Bacteroidetes and the class Bacteroida were detected among the groups, the abundance of an unidentified bacteria belonging to the family *Weeksellaceae* (Class Bacteroida, Order Flavobacteriales) was incremented in C/Fp (C/Fp: 3.5 ± 1.9%; C: 1.0 ± 0.8%; adjusted *P* < 0.05). Finally, a significant decrease in the phylum Cyanobacteria reflected in the class Oxyphotobacteria was observed in PS/Fp compared to PS (PS: 2.0 ± 1.8%; PS/Fp: 0.02 ± 0.1%; adjusted *P* = 0.0346). However, these variations may be due to the presence of algae in the samples as a significant increment of the “*o*_*Chloroplast_OTU_27*” were detected in PS (adjusted *P* = 0.0322).

### Changes in the Composition After the Bacterial Infection and in Relation to Antibiotic Administration (Microbial Gut Community 8 dpi)

Two dpi and for 10 consecutive days, the antibiotic florfenicol was administered in An and An/Fp. Eight dpi, fish in C, An, C/Fp, and An/Fp were sampled. At this time point, 80 and 0% of sampled fish in C/Fp and An/Fp were positive to *F. psychrophilum*, respectively (Figure 1). Sampled fish in C and An were negative to the bacterium.

**FIGURE 7:**
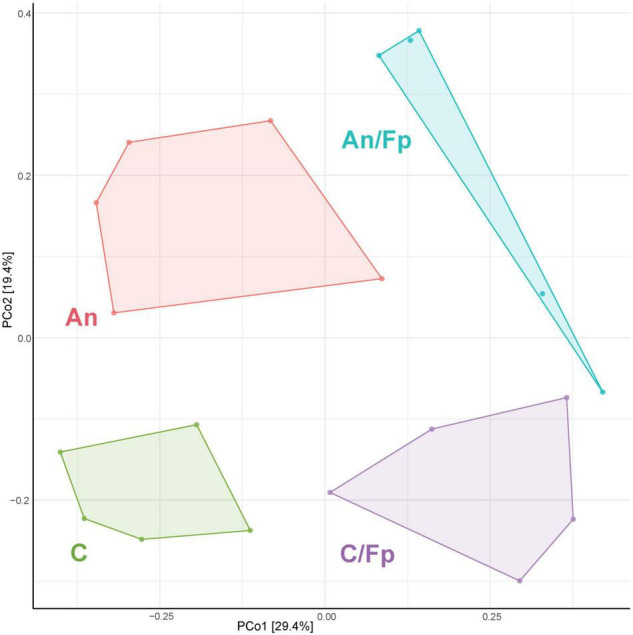
PCoA of 20 samples and 310 OTUs (8 dpi) [The OTUs were transformed to relative abundance]. Prior to the analysis, OTU’s that are not present in more than 0.1% relative abundance in any sample have been removed. No initial data transformation has been applied. The relative contribution (eigenvalue) of each axis to the total inertia in the data is indicated in percent at the axis titles. C, control feed group; An, antibiotic feed group; C/Fp, control feed group + *F. psychrophilum*; An/Fp, antibiotic feed group + *F. psychrophilum*.

The PCoA analysis (Figure 7) showed a clear shift in the microbial composition of the gut during the bacterial infection (C/Fp and An/Fp) and in relation to the antibiotic treatment (An and An/Fp) compared to fish in C. Indeed, four separated independent clusters can be observed (*P* = 0.001, PERMANOVA).

**FIGURE 8:**
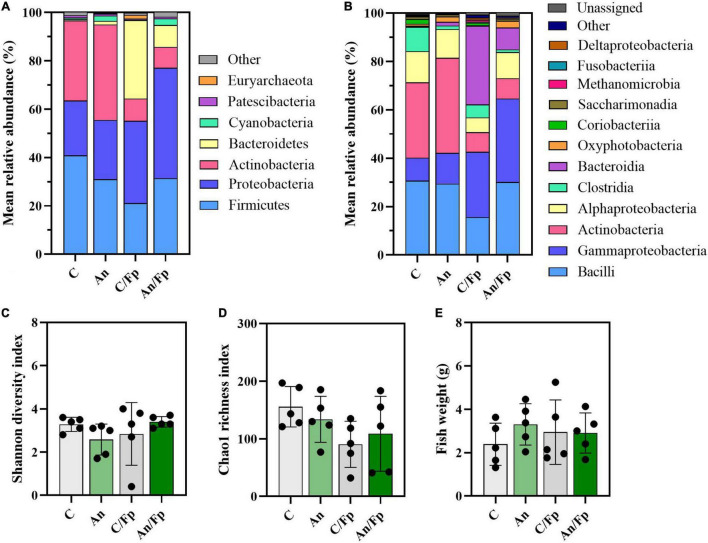
Microbial community (8 dpi). Characterization of mean relative abundance at phylum **(A)** and class **(B)** level, Shannon diversity index **(C)**, Chao1 richness index **(D)**, and fish weight **(E)**. Values represent the mean of five samples. In **(C–E)**, error bars represent the standard deviation. Shannon diversity index values are based on 10,000 reads per sample. C, control feed group; An, antibiotic feed group; C/Fp, control feed group + *F. psychrophilum*; An/Fp, antibiotic feed group + *F. psychrophilum*.

**FIGURE 9:**
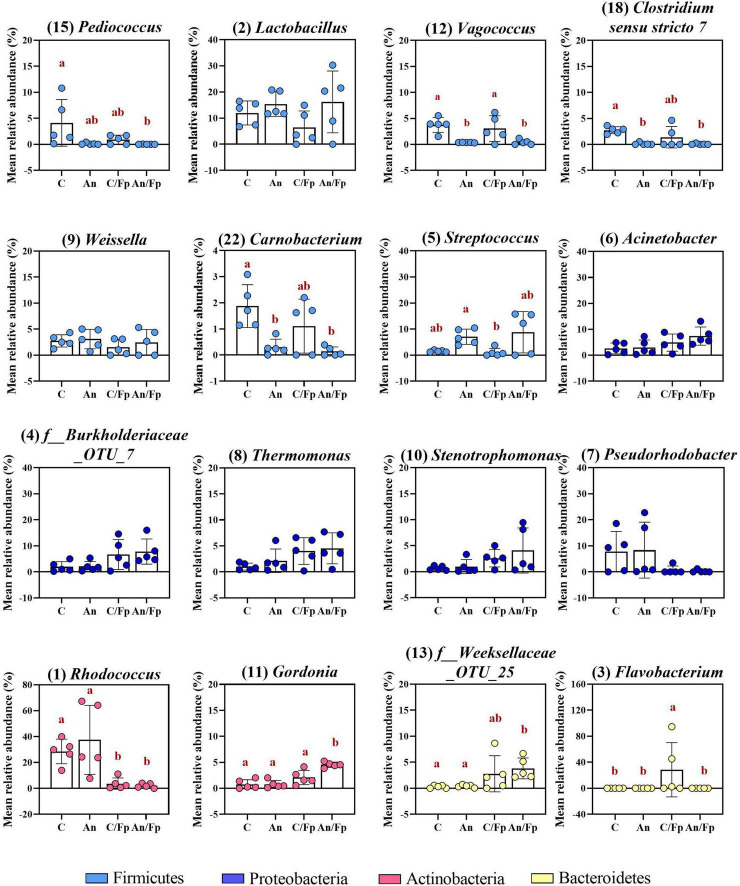
Top-13 most abundant, plus three additional genera, selected among the top-30 in the control and antibiotic feed groups with (C/Fp and An/Fp) and without (C and An) bacterial exposure (8 dpi). The selection of the additional genera was based on previously observed abundances (among top-13 at day -1). The number in parenthesis states the position of the genus in the ranking. Values represent the mean and SD of five samples. Differences are tested by ANOVA or Kruskal–Wallis (*P*-values are adjusted for multiple comparison). When significant, differences are presented with different red letters. See Supplementary Table 10 for more information on the top-30 most abundant genera at day 8.

Dissimilarities were also revealed by the taxonomic mapping (Figures 8, 9 and Supplementary Tables 8–10). However, no statistically significant differences in the Shannon diversity index (C: 3.3 ± 0.3), the Chao1 richness index (C: 155.7 ± 35.1), and the fish size (C: 2.4 ± 1.0 g) were observed between the groups (Figures 8C–E). A large variation in the Shannon diversity index was detected for C/Fp (2.8 ± 1.5) and in the Chao1 richness index for An/Fp (108.8 ± 65.0).

The antibiotic administration caused variation in the abundance of bacteria within the phylum Firmicutes. Differently from the previous time points, variations in the genera *Streptococcus*, *Vagococcus, Carnobacterium* (Phylum Firmicutes; Class Bacilli; Order Lactobacillales), and *Clostridum sensu stricto 7* (Phylum Firmicutes; Class Clostridia; Order Clostridiales) were recorded in An and An/Fp compared to C and C/Fp (Figure 9 and Supplementary Table 10). The genus *Streptococcus* was more copious in An (7.1 ± 2.9%) and An/Fp (8.8 ± 7.9%) (An/Fp vs. C/Fp: adjusted *P* = 0.0333). The genera *Vagococcus, Carnobacterium*, and *Clostridum sensu stricto 7* were instead strongly reduced as their values were close to zero in An and An/Fp (adjusted *P* < 0.05). In addition, other differences can be observed within the Firmicutes compared to the previous time points. The reduced abundance of the genus *Lactobacillus* observed 1 dpi in An/Fp compared to C was not observed 8 dpi, as well as no difference, was recorded in the genus *Weissella* (at day -1 and 1 dpi, it was reduced in An and An/Fp). The genus *Pediococcus* dropped to zero in An, C/Fp, and An/Fp. In C/Fp, these values were already observed 1 dpi. Unlike the high abundance of the genus *Staphylococcus* detected in An at day -1 and the 1-2% abundance detected 1 dpi for all groups, its abundance was ∼ 0% 8 dpi (data not presented).

The bacterial infection induced changes among the phylum Proteobacteria as a general increase in the class Gammaproteobacteria was detected in infected fish (C: 9.5 ± 2.25%; An: 12.7 ± 12.5%; C/Fp: 27.3 ± 17.5%; An/Fp: 34.4 ± 15.9%) (C vs. An/Fp: adjusted *P* = 0.0274). However, no significant difference was observed at the genus level among the most abundant bacteria belonging to this class. This was different from what was observed 1 dpi, where the genus *Thermomonas* was significantly increased in C/Fp compared to C (Figures 8, 9 and Supplementary Tables 8–10).

The effects of the bacterial infection were also observed in other phyla (Figure 8 and Supplementary Tables 8, 9). Indeed, the bacterial challenged fish were also characterized by an inferior abundance of the phylum Actinobacteria compared to non-challenged fish (C: 33.1 ± 7.9%; An: 39.4 ± 26.0%; C/Fp: 9.2 ± 7.7%; An/Fp: 8.6 ± 1.5%) (adjusted *P* < 0.05), and by a significant rise of the phylum Bacteroidetes when not fed with the antibiotic-coated feed (C: 0.5 ± 0.6%; C/Fp: 32.6 ± 39.3%; adjusted *P* = 0.008). These changes were reflected in the class Actinobacteria and Bacteroida. At genus level (Figure 9 and Supplementary Table 10), the mean abundances of *Rhodococcus* and *Gordonia* (phylum Actinobacteria) were affected by the infection. The genus *Rhodococcus* dropped to zero in C/Fp and An/Fp, similarly to what was observed 1 dpi, compared to C (28.5 ± 9.5%) and An (37.4 ± 26.7%) (adjusted *P* < 0.05). The abundance of this genus in C was higher at this time point than what was observed in C 1 dpi (Mann–Whitney test; *P* = 0.0079). The rise in the genus *Gordonia* in C/Fp observed 1 dpi was lost. However, a significant increase was detected in An/Fp (4.5 ± 0.5%) compared to the other groups (adjusted *P* ≤ 0.02). The genus *Flavobacterium* (Class Bacteroida, Order Flavobacteriales) became bigger in C/Fp (28.5 ± 41.7%; adjusted *P* ≤ 0.02) as, probably, a direct consequence of the higher percentage of detection of *F. psychrophilum* in C/Fp sampled fish 8 dpi (80% against 0% in An/Fp). Among the Class Bacteroida, the abundance of an unidentified bacteria belonging to the family *Weeksellaceae* was also affected by the infection, as higher values were recorded in C/Fp and An/Fp, similarly to what observed 1 dpi.

Similarly as observed 1 dpi for PS, an increased abundance within the phylum Cyanobacteria and the class Oxyphotobacteria was recorded in fish subjected to the antibiotic therapy (An: 2.3 ± 1.4%; An/Fp: 2.7 ± 2.0%; adjusted *P* ≤ 0.02 vs. C/Fp) (Figure 8 and Supplementary Tables 8, 9). In this case, these variations may be due to the presence of algae in the samples as a significant increment of the “*o*_*Chloroplast_OTU_35*” was detected (adjusted *P* < 0.05) (Supplementary Table 10).

### Gut Microbial Community of Rainbow Trout Fry 33 dpi: Changes in the Composition After Recovery From the Bacterial Infection and in Relation to Antibiotic or Phage Administration

To assess the composition of the gut microbial community after recovery from the bacterial infection and in relation to antibiotic and phage administration, fish were sampled 33 dpi (all negative to *F. psychrophilum*) (Figure 1).

**FIGURE 10:**
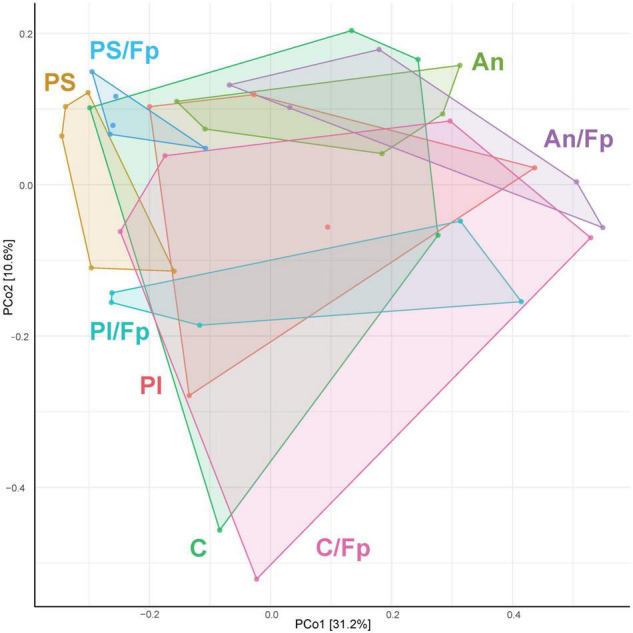
PCoA of 40 samples and 448 OTUs (33 dpi) [The OTUs were transformed to relative abundance]. Prior to the analysis, OTU’s that are not present in more than 0.1% relative abundance in any sample have been removed. No initial data transformation has been applied. The relative contribution (eigenvalue) of each axis to the total inertia in the data is indicated in percent at the axis titles. C, control feed group; An, antibiotic feed group; PI, phage-immobilized feed group; PS, phage-sprayed feed group; C/Fp, control feed group + *F. psychrophilum*; An/Fp, antibiotic feed group + *F. psychrophilum*; PI/Fp, phage-immobilized feed group + *F. psychrophilum*; PS/Fp, phage-sprayed feed group + *F. psychrophilum.*

As performed in the other time points, the microbial communities of the fish gut were visualized in a PCoA plot (Figure 10). No statistically significant difference was observed in the microbial composition in the groups that have been subjected to the bacterial infection in comparison to the non-challenged groups (*P* = 0.135, PERMANOVA). Among the non-challenged fish, the gut microbial communities in the four groups were significantly different (*P* = 0.003, PERMANOVA), even if the microbiome of fish in C seemed to be fairly diverse. No differences were recorded between C and An (*P* = 0.115, PERMANOVA) while PI and PS were statistically different between each other (*P* = 0.025, PERMANOVA) and in comparison with C and An (*P* = 0.045, PERMANOVA). A similar pattern was observed in fish that had recovered the infection in the four groups (*P* = 0.017, PERMANOVA). No differences were recorded between C/Fp and An/Fp (*P* = 0.241, PERMANOVA), while PI/Fp and PS/Fp were statistically different between each other (*P* = 0.032, PERMANOVA) and in comparison with C/Fp and An/Fp (*P* = 0.014, PERMANOVA). In addition, fish in PS and PS/Fp were characterized by a smaller in-group variation compared to the other groups.

**FIGURE 11:**
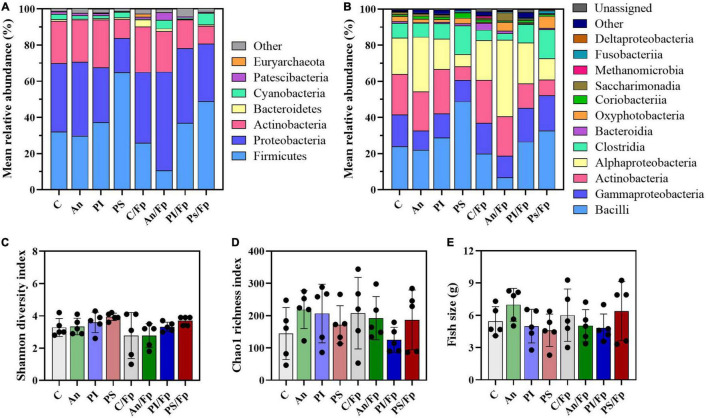
Microbial community 33 dpi. Characterization of mean relative abundance at phylum **(A)** and class **(B)** level, Shannon diversity index **(C)**, Chao1 richness index **(D)** and fish weight **(E)**. Values represent the mean of five samples. In **(C,D)**, error bars represent the standard deviation. Shannon diversity index values are based on 10,000 reads per sample. C, control feed group; An, antibiotic feed group; PI, phage-immobilized feed group; PS, phage-sprayed feed group; C/Fp, control feed group + *F. psychrophilum*; An/Fp, antibiotic feed group + *F. psychrophilum*; PI/Fp, phage-immobilized feed group + *F. psychrophilum*; PS/Fp, phage-sprayed feed group + *F. psychrophilum*.

The taxonomic mapping did not reveal dissimilarities at phylum (top-5) and class (top-6) level except that a higher abundance of Cyanobacteria reflected in the class Oxyphotobacteria was detected in the group PS/Fp (6.4 ± 3.0%) compared to C/Fp (0.5 ± 0.5%) (Figures 11A,B and Supplementary Tables 11, 12). Similarly as the previous time points, these variations may be due to the presence of algae in the samples, e.g., a significant increment of the “*o*_*Chloroplast_OTU_27*” (adjusted *P*-value PS/Fp vs. C/Fp = 0.0254) was detected. The Shannon diversity index, the Chao1 richness index, and fish size are presented in Figures 11C–E, respectively. No statistically significant difference was observed between the groups likely due to the large variation observed between replicates.

**FIGURE 12:**
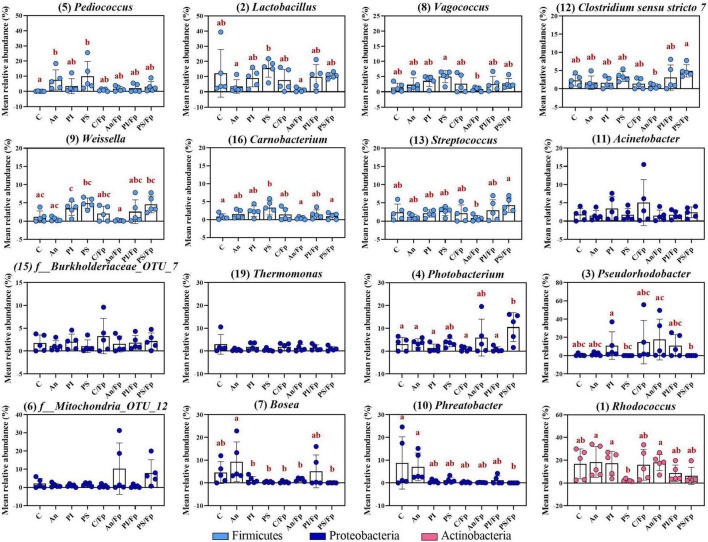
Top-13 most abundant, plus three additional genera, selected among the top-30 33 dpi in fish fed with control, antibiotic, phage-immobilized and phage-sprayed feed with (C/Fp; An/Fp; PI/Fp; PS/Fp) and without (C; An; PI; PS) bacterial exposure. The selection of the additional genera was based on previously observed abundances (among top-13 at day -1). The number in parenthesis states the position of the genus in the ranking. Values represent the mean and SD of five samples. Differences are tested by ANOVA or Kruskal–Wallis (*P*-values are adjusted for multiple comparison). When significant, differences are presented with different red letters. See Supplementary Table 13 for more information on the top-30 most abundant genera at day 33.

The top-30 most abundant genera are shown in Figure 12 and Supplementary Table 13. Among the Firmicutes, some of the previously observed differences were restored. In contrast to what was observed 8 dpi, the abundance of the genera *Vagococcus*, *Streptococcus*, and *Clostridium sensu stricto 7* were restored in An and An/Fp as no significant difference was detected with C and C/Fp (adjusted *P* > 0.05). Also, the reduced abundances compared to C of *Vagococcus* in PI and PS/Fp, and of *Lactobacillus* in PI, An/Fp, and PS/Fp observed 1 dpi were also re-established. Nevertheless this trend, new differences among the feed types were detected: the genera *Pediococcus* and *Carnobacterium* were increased in PS (*Pediococcus*: 10.0 ± 9.7%; *Carnobacterium*: 3.4 ± 2.0%) compared to fish fed C (*Pediococcus*: 0.1 ± 0.1%; *Carnobacterium*: 0.8 ± 0.8%) (adjusted *P*: *Pediococcus* = 0.0012*; Carnobacterium* = 0.0264). Finally, similar to what was observed at day -1 and 1 dpi, the genus *Weissella* was characterized by the lowest values in An and An/Fp. However, no statistically significant difference was measured with C and C/Fp. Unlike the high abundance of the genus, *Staphylococcus* detected in An at day -1 and the 1–2% abundance detected 1 dpi for all groups, its abundance was ∼0% as at 8 dpi (data not presented).

Similarly as for bacteria belonging to the phylum Firmicutes, some of the differences observed in Proteobacteria at the previous time points disappeared. For example, the increased abundance of *Thermomonas* detected in C/Fp and PS/Fp measured 1 dpi was lost as no significant difference was detected 33 dpi. A similar pattern was observed for the genera *Stenotrophomonas* and *Acinetobacter.* However, new dissimilarities were measured: the genus *Pseudorhodobacter* (previously only observed in few replicates; no significant differences detected at day -1 and, 1 and 8 dpi) was significantly increased in PI and An/Fp (compared to PS and PS/Fp; adjusted *P* < 0.05) and characterized by a large variation in the feed groups C/Fp and PI/Fp; the genus *Photobacterium* (significantly increased in PS 1 dpi) was more copious in the group PS/Fp (10.5 ± 6.3%) compare to C (adjusted *P* = 0.0306); a higher abundance of the genus *Phreatobacter* was detected in the groups C (8.8 ± 11.5%) and An (7.0 ± 6.1%) compared to PS/Fp (adjusted *P* < 0.05). Differently than what observed at day -1, no significant differences were observed in the genus *Enhydrobacter* between PS and the other feed groups.

Among the phylum Actinobacteria, the genus *Rhodococcus* dropped to 2.3 ± 1.1% in PS compared to An (18.4 ± 13.6%) and PI (17.2 ± 10.4%) (adjusted *P* < 0.05). Similarly as 8 dpi, the abundance of this genus in C was higher 33 dpi than what observed in C 1 dpi (Mann–Whitney test; *P* = 0.0317). Dissimilarities in the genus *Gordonia* observed 1 and 8 dpi were lost (0–1% in all groups). Finally, previously observed dissimilarities of the genus *Flavobacterium* (∼0%) and an unidentified bacteria belonging to the family *Weeksellaceae* (*f_Weeksellaceae_OTU_25*) (0–1%) were not observed.

## Discussion

To study the microbial composition of the gut of rainbow trout fry exposed to phage and antibiotic therapies, the intestine including the gut content (if present) [necessitating mixing of autochthonous and allochthonous communities] of healthy and infected fish exposed to the different feed regimes was sampled.

### Microbial Gut Community of Rainbow Trout Fry (Day -1)

The gut microbiota of fish sampled before performing the bacterial challenge was characterized by bacteria belonging to the phyla Firmicutes (∼50%) and Proteobacteria (∼20%), which were the most abundant, followed by Actinobacteria (∼10%), Bacteroidetes (∼2%) and Cyanobacteria (∼1%). These results are in accordance with previous research focused on the gut autochthonous microbial composition of rainbow trout at early life stages fed with marine diets (Inicio plus not including the yeast-based additive, BioMar A/S, Brande, Denmark) (Ingerslev et al., 2014a,b) but also with the general composition of the gut microbiota of teleost (reviewed by Tarnecki et al., 2017; Egerton et al., 2018). In addition to these phyla, a higher abundance of Tenericutes and Proteobacteria have been detected in the distal content of rainbow trout juveniles (Lyons et al., 2017; Villasante et al., 2019).

When looking at the genus level, the genera *Pediococcus*, *Lactobacillus*, *Vagococcus* (belonging to the phylum Firmicutes), *Acinetobacter*, *Thermomonas* (phylum Proteobacteria), and *Rhodococcus* (phylum Actinobacteria) were the most abundant. The abundance of the genus *Pediococcus* might be linked to the presence of the probiotic lactic acid bacterium *Pediococcus acidilactici* (Bactocell^®^) in the selected commercial feed. Thus, differences in the abundance of this genus during the experiment may be related to the impaired feed intake normally observed in diseased fish.

### Changes in the Composition After the Bacterial Infection and in Relation to Antibiotic Administration

Infection and antibiotic therapy are known factors able to alter the composition of the gut microbiota (reviewed in teleost by Butt and Volkoff, 2019). In our experiment, the effects of the infection in fish fed control feed (C/Fp) were observed 1 and 8 dpi as dissimilarities were revealed by the β-diversity analysis and by the taxonomical mapping. Alpha diversity measures were not affected by the infection even if lower (but not statistically significant) Chao1 richness index values were recorded for fish exposed to *F. psychrophilum* 8 dpi. It is important to mention that the bacteriological examination showed we were able to re-isolate *F. psychrophilum* in 60 and 80% of the fish in the control feed group 1 and 8 dpi (C/Fp), respectively [a low infection dose was selected for the experiment (1*10^4^ CFU fish^–1^) to increase the MOI]. We decided to not remove infected fish, where the pathogen was not re-isolated at these time points from the analysis, since we knew that all fish were inoculated with the bacteria even though the level of infection varied among individuals.

In our study, the taxonomic mapping revealed that the infection caused by *F. psychrophilum* altered the ratio Firmicutes/Proteobacteria. Indeed, a decrease in Firmicutes (at genus level: *Lactobacillus* 1 dpi) and a rise in the Proteobacteria (at class level: Gammaproteobacteria 1 and 8 dpi; at genus level: *Thermomonas* 1 dpi) were observed. Previous experiments investigating the effects of *Yersinia ruckeri* infection on the autochthonous gut microbial community of rainbow trout fry were performed by Ingerslev et al. (2014a) and changes in the bacterial abundances were observed in challenged fish. For example, an increase in Proteobacteria reflected in a higher number of bacteria belonging to the genus *Aeromonas* (causing a decrease in the abundance of Firmicutes) was observed 19 dpi with *Y. ruckeri* (the genus *Aeromonas* contains opportunistic pathogens for fish).

Actinobacteria are Gram-positive non-motile rods, mainly anaerobic (Rizzatti et al., 2017) and, in our experiment, their abundance was impaired by the infection, i.e., the genus *Rhodococcus* was lower in infected fish (1 and 8 dpi) while the genus *Gordonia* increased (highest abundance when combined with antibiotic administration 8 dpi). Bacteria belonging to the genus *Rhodococcus* (one of the most abundant genera observed in this study) have been detected in the gut microbiome of rainbow trout fry also by Ingerslev et al. (2014a,b). This genus includes Gram-positive cocci/rod-shaped anaerobic bacteria (Walsh et al., 1993) and some studies have looked into using *Rhodococcus* spp. as probiotics in aquaculture (e.g., for prevention of bacterial infections) (Boutin et al., 2013; Sharifuzzaman et al., 2018). Thus, the infection caused by *F. psychrophilum* clearly affects a population of bacteria that could be beneficial for the fish. Concerning the increased abundance of *Gordonia* (phylum Actinobacteria), bacteria belonging to this genus (suborder *Corynebacterineae*) are actinomycetes containing mycolic acid (long-chain fatty acids) and of biotechnological interest because of the various range of chemical compounds they produced. However, some are considered opportunistic pathogens (Arenskötter et al., 2004).

Florfenicol is a broad-spectrum antibiotic (sensitive bacteria include Gram-negative bacilli, Gram-positive cocci, and other bacteria such as mycoplasma; principle of action: inhibition of protein synthesis) (Papich(ed.), 2016) that has been used in Danish fish farms since 1996 to control RTFS (Bruun et al., 2000). Antibiotics with a wide broad-spectrum are known to cause alternations in the bacterial community (e.g., reduced microbial diversity, increased chance that opportunistic pathogens proliferate) and various studies have been targeting this topic in teleosts (Gupta et al., 2019; Kokou et al., 2020). In our study, the gut microbial community was affected by the antibiotic therapy as revealed by the β-diversity analysis and the taxonomic mapping 8 dpi. In contrast, measures of α-diversity were not. These results are in line with what was observed by Kokou et al. (2020) in seabass (study of microbiota of the pyloric caeca, mid-and hindgut). Other studies, however, have recorded a higher α-diversity after antibiotics treatment. Gupta et al. (2019) studied the autochthonous communities of the gut of Atlantic salmon after the administration of florfenicol and oxolinic acid and observed a higher α-diversity in the distal part of the intestine following florfenicol administration. No increase was observed in the case of oxolinic acid treatment.

The immediate effect of florfenicol was observed in relation to the genus *Flavobacterium*, which was not detected among the most abundant genera in infected fish fed with antibiotics suggesting that 8 dpi fish had already recovered (0% of fish in An/Fp were positive to the bacterium). Indeed, this genus was highly increased in challenged fish fed control feed (80% of fish in C/Fp were positive to the bacterium 8 dpi). Further, when looking at the taxonomic mapping during the administration of florfenicol, we recorded variations in the genera *Streptococcus*, *Vagococcus, Carnobacterium* (class Bacilli, phylum Firmicutes), *Clostridium sensu strictu 7* (class Clostridia, phylum Firmicutes) and, as already mentioned in combination with the infection, *Gordonia* (class Actinobacteria, phylum Actinobacteria). Changes in the microbial abundances related to antibiotic administration were also observed by Gupta et al. (2019) and Kokou et al. (2020), which noticed that the tested antibiotics affected more markedly the composition of the distal gut microbiota than the midgut community. In Kokou et al. (2020), the authors also observed that antibiotics with the same principle of action can affect the gut microbial communities in different manners (e.g., in how broad they are).

*Streptococcus* and *Vagococcus* are lactic acid bacteria (LAB) (Gram-positive cocci) performing homolactic fermentation that have been associated with the commensal gut microbiota of salmonids (Ringø and Gatesoupe, 1998; Ingerslev et al., 2014a,b). An increased abundance of these genera has been associated with plant-based diets and suggested to be beneficial for the fish immune system since these bacteria may help to protect from pathogens that could penetrate the intestinal barrier (Ingerslev et al., 2014a,b). In our experiment, we observed a decrease in the number of bacteria belonging to the genus *Vagococcus* during florfenicol administration suggesting that the treatment may negatively affect part of the beneficial bacterial community. The number of bacteria belonging to the genus *Streptococcus* was instead increased. One should remember that this genus as well as other LAB also contains pathogenic bacteria (Ringø and Gatesoupe, 1998).

The gut microbial community is malleable and able to recover from infections and antibiotics treatment (Francino, 2016). In our study, previously observed dissimilarities between the microbial community of non-challenged and recovered fish were lost 33 dpi. No statistically significant difference was detected in the β-diversity analysis (+Fp vs. –Fp). Also, the taxonomic mapping revealed the previously observed differences in the genera *Streptococcus*, *Vagococcus*, *Carnobacterium*, *Clostridium sensu strictu 7* (Firmicutes), *Rhodococcus*, *Gordonia* (Actinobacteria), *Flavobacterium*, and the unidentified bacterium belonging to the family *Weeksellaceae* (Bacteroidetes) were recovered.

### Changes in the Composition in Relation to Phage Administration in Healthy and Infected Rainbow Trout Fry

Bacteriophages are species-specific viruses of bacteria and they are studied in gut microbiome therapy research to target specific gut pathogens and so restore beneficial bacteria (Zheng et al., 2019; Dahlman et al., 2021). Since they target specific bacterial populations, lytic phages are generally considered not able to alter the intestinal bacterial communities. However, these considerations are being revised/discussed as new studies have demonstrated, at different levels of analysis (alpha- or beta-diversity and taxonomic mapping), the ability of lytic phages to alter the gut microbiome independently of the presence of their target bacteria (Silva et al., 2016; Tetz et al., 2017; Barr, 2019; Febvre et al., 2019; Hsu et al., 2019). Other studies have instead not revealed changes in the gut microbiome as a result of phage therapy (Richards et al., 2019).

In the current work, we were not expecting any significant change in the overall gut microbiome of fish exposed to phages by oral administration, since our phages were specific for the freshwater pathogen *F. psychrophilum* (not considered part of the normal microbiome in high abundances). However, this was not the case as after 11 days of phage administration via phage-immobilized or phage-sprayed feed (day -1), differences in the gut microbial composition compared to the control groups were revealed by the β-diversity analysis, and the same pattern was observed 1 and 33 dpi, independently if the fish were exposed or not to *F. psychrophilum*. Further, differences between PI and PS were observed at a different degree at the selected time points, more evidently 33 dpi. This may be due to the two different application methods chosen for this experiment (PI: corona-discharge technology that covalently binds phages to feed pellets; PS: phage solution sprayed on pellets). When we analyzed the abundance of phages in the intestine, we observed a very similar and stable phage number. However, a lower phage translocation efficiency was observed in the internal organs of fish feed PI. It is not clear if this was due to a lower number of phages attached to the feed pellets or a tighter binding of the phages to the pellets (Donati et al., 2021b).

The reasons for these effects of phages on gut community composition may relate to indirect effects of phage infections of *F. psychrophilum*. The release of cell lysates from infected cells has been shown to favor specific bacteria (Middelboe et al., 2003), and may thus affect the composition of the microbiota. Alternatively, other phage-susceptible Flavobacterial populations may have been present in the gut. Also, it has been hypothesized that phages may evolve to become able to infect other bacteria than their original target (reviewed by Ganeshan and Hosseinidoust, 2019). Overall, the delivery of a high quantity of specific phages may disturb the interactions between the phage and bacteria populations directly or indirectly.

The observed alteration in the overall population identified by the β-diversity analysis was not revealed by the taxonomic mapping, suggesting that the administration of phages was mostly influencing the richness of low abundant bacteria and/or the dynamics between gut bacteria and phage populations (i.e., different bacteria were enhanced or decreased at different time points in non-infected fish fed phage-treated feed). One day before the infection, the taxonomic mapping revealed that dissimilarities at the genus level, as significantly higher abundances of the genera *Enhydrobacter* and *Stenotrophomonas* (phylum Proteobacteria), were detected for fish fed phage-sprayed feed and for both phage-treated feed types, respectively (compared to the control). The higher abundance of *Stenotrophomonas* was maintained in fish-fed phage-immobilized feed 1 dpi (non-infected fish; but not at 33 dpi), while the differences in *Enhydrobacter* were not further observed. Another example is the enhanced abundances of various LAB 33 dpi by phage-treated feed, more markedly for phage-sprayed feed (non-infected fish) (not previously observed). Among them, we found the genus *Carnobacterium*. Bacteria belonging to this genus have been tested as probiotics against various fish bacterial infections (Ringø et al., 2010).

These observed changes in the gut microbiota in response to phage-treated feed administration did not affect the fish growth and no negative fish health parameters were recorded (Donati et al., 2021b). Consequently, even though the selected phages did affect the gut microbiome, no negative implications were observed. However, further research should be targeting, e.g., the metabolome (= collection of metabolites that provides a direct readout of cellular activity; Sun and Hu, 2016) and the immune response that phages can trigger as a limited number of studies have been conducted about phage therapy and their effects on the immune response in fish (e.g., Schulz et al., 2019a,b).

## Conclusion

The gut microbiota composition of rainbow trout fry observed in this study is in line with what was previously observed. The bacterial infection and the antibiotic administration caused changes in the microbial composition of the gut, which were then lost once fish recovered from the infection and the antibiotic treatment was terminated. Interestingly, the administered phages changed the overall composition of the gut microbiota independently of the infection. Thus, future studies should try to resolve the mechanism of phage-driven changes in the microbiota and understand how they impact the immune response of the fish.

## Data Availability Statement

The datasets presented in this study can be found in online repositories. The names of the repository/repositories and accession number(s) can be found below: NCBI BioProject PRJNA786762 (http://www.ncbi.nlm.nih.gov/bioproject/786762 and https://www.ncbi.nlm.nih. gov/sra/PRJNA786762).

## Ethics Statement

The animal study was reviewed and approved by the Animal Experiments Inspectorate of Denmark (Dyreforsøgstilsynet, permission numbers 2013-15-2934-00976 until 07.10.2019 and 2019-15-0201-00159 from 08.10.2019).

## Author Contributions

VD: planning and execution of fish experiments, fish sampling, DNA extraction, data analysis and visualization, and writing of the manuscript. LM and ID: planning, execution and supervision of fish experiments, data interpretation, and manuscript preparation. MM: data interpretation, manuscript preparation, and funding acquisition. MS: planning, data interpretation, and manuscript preparation. All authors read and approved the final version of the manuscript.

## Conflict of Interest

The authors declare that the research was conducted in the absence of any commercial or financial relationships that could be construed as a potential conflict of interest. The handling editor JL declared a shared affiliation with the author MM at the time of review.

## Publisher’s Note

All claims expressed in this article are solely those of the authors and do not necessarily represent those of their affiliated organizations, or those of the publisher, the editors and the reviewers. Any product that may be evaluated in this article, or claim that may be made by its manufacturer, is not guaranteed or endorsed by the publisher.
