# Supplementary material for: The Gut Microbiota of Healthy and Flavobacterium psychrophilum-Infected Rainbow Trout Fry Is Shaped by Antibiotics and Phage Therapies
Source: Front Microbiol. 2022 May 10;13:771296. doi: 10.3389/fmicb.2022.771296 (PMC9128845; doi:10.3389/fmicb.2022.771296)
Supplement: Supplementary file 1 [file Data_Sheet_1.pdf]

Supplementary material

Supplementary Table 1. Characteristics of sequenced samples. C: control group; An: antibiotic feed group; PI: phage-immobilized feed group; PS: phage-sprayed feed group. Red: failed sequencing; yellow: low output samples; green: highest number of reads obtained for one sample. Dpi: days post infection.

Feed group	Bacterial infection	Re-isolation of *F. psychrophilum*	Dpi	Sample no.	Number of reads	Observed OTUs
C	NO	NO	-1	1	51143	142
C	NO	NO	-1	2	57811	183
C	NO	NO	-1	3	52746	104
C	NO	NO	-1	4	43455	122
C	NO	NO	-1	5	55967	120
C	NO	NO	1	1	26690	110
C	NO	NO	1	2	19515	89
C	NO	NO	1	3	39887	205
C	NO	NO	1	4	20454	112
C	NO	NO	1	5	25004	131
C	NO	NO	8	1	34420	176
C	NO	NO	8	2	38885	178
C	NO	NO	8	3	29163	118
C	NO	NO	8	4	30443	121
C	NO	NO	8	5	36302	125
C	NO	NO	33	1	4624	43
C	NO	NO	33	2	35193	236
C	NO	NO	33	3	45460	154
C	NO	NO	33	4	29804	75
C	NO	NO	33	5	41789	181
C	YES	NO	1	1	27126	247
C	YES	YES	1	2	27076	190
C	YES	YES	1	3	24700	126
C	YES	NO	1	4	16905	116
C	YES	YES	1	5	32112	246
C	YES	NO	8	1	20236	116
C	YES	YES	8	2	19638	87
C	YES	YES	8	3	28374	31
C	YES	YES	8	4	14904	61
C	YES	YES	8	5	24858	130
C	YES	NO	33	1	40387	210
C	YES	NO	33	2	6520	43
C	YES	NO	33	3	39938	150
C	YES	NO	33	4	41062	259
C	YES	NO	33	5	36001	217
An	NO	NO	-1	1	48950	136
An	NO	NO	-1	2	56185	200
An	NO	NO	-1	3	21620	100
An	NO	NO	-1	4	49981	136
An	NO	NO	-1	5	58179	164
An	NO	NO	1	1	24880	143
An	NO	NO	1	2	17206	84
An	NO	NO	1	3	22151	100
An	NO	NO	1	4	37486	188
An	NO	NO	1	5	6492	40
An	NO	NO	8	1	48384	177
An	NO	NO	8	2	26360	122
An	NO	NO	8	3	27416	113

Feed group	Bacterial infection	Re-isolation of *F. psychrophilum*	Dpi	Sample no.	Number of reads	Observed OTUs
An	NO	NO	8	4	41703	146
An	NO	NO	8	5	17010	63
An	NO	NO	33	1	37556	189
An	NO	NO	33	2	22129	207
An	NO	NO	33	3	34473	250
An	NO	NO	33	4	23269	119
An	NO	NO	33	5	32966	239
An	YES	NO	1	1	27299	107
An	YES	YES	1	2	24421	58
An	YES	NO	1	3	18268	107
An	YES	YES	1	4	30170	146
An	YES	YES	1	5	26023	111
An	YES	NO	8	1	13908	41
An	YES	NO	8	2	39186	148
An	YES	NO	8	3	38832	161
An	YES	NO	8	4	32874	118
An	YES	NO	8	5	7944	41
An	YES	NO	33	1	46642	276
An	YES	NO	33	2	35065	209
An	YES	NO	33	3	31231	146
An	YES	NO	33	4	44012	149
An	YES	NO	33	5	48113	129
PI	NO	NO	-1	1	25820	120
PI	NO	NO	-1	2	31213	154
PI	NO	NO	-1	3	9843	49
PI	NO	NO	-1	4	35043	133
PI	NO	NO	-1	5	19509	101
PI	NO	NO	1	1	5013	33
PI	NO	NO	1	2	10610	37
PI	NO	NO	1	3	437110	244
PI	NO	NO	1	4	13322	51
PI	NO	NO	1	5	31065	158
PI	NO	NO	33	1	41832	275
PI	NO	NO	33	2	31321	115
PI	NO	NO	33	3	30043	248
PI	NO	NO	33	4	43626	248
PI	NO	NO	33	5	15936	79
PI	YES	YES	1	1	15344	63
PI	YES	YES	1	2	24950	112
PI	YES	YES	1	3	21601	76
PI	YES	NO	1	4	32256	131
PI	YES	YES	1	5	36352	140
PI	YES	NO	33	1	34263	136
PI	YES	NO	33	2	37301	171
PI	YES	NO	33	3	21890	76
PI	YES	NO	33	4	19135	77
PI	YES	NO	33	5	35283	87
PS	NO	NO	-1	1	34055	146
PS	NO	NO	-1	2	11940	57
PS	NO	NO	-1	3	22176	196
PS	NO	NO	-1	4	22972	107
PS	NO	NO	-1	5	FAILED	FAILED
PS	NO	NO	1	1	9573	53
PS	NO	NO	1	2	17074	77
PS	NO	NO	1	3	18206	108

**Feed group**	**Bacterial infection**	**Re-isolation of *F. psychrophilum***	**Dpi**	**Sample no.**	**Number of reads**	**Observed OTUs**
PS	NO	NO	1	4	32840	135
PS	NO	NO	1	5	26061	176
PS	NO	NO	33	1	32386	165
PS	NO	NO	33	2	22952	110
PS	NO	NO	33	3	22403	130
PS	NO	NO	33	4	31773	158
PS	NO	NO	33	5	29542	254
PS	YES	YES	1	1	13236	38
PS	YES	YES	1	2	23924	132
PS	YES	YES	1	3	5407	33
PS	YES	YES	1	4	8158	36
PS	YES	YES	1	5	19737	39
PS	YES	NO	33	1	22432	84
PS	YES	NO	33	2	31855	215
PS	YES	NO	33	3	19640	248
PS	YES	NO	33	4	23998	75
PS	YES	NO	33	5	29967	234

Supplementary Table 2. Top five most abundant phyla at day -1 in the feed groups (note that fish in the control and antibiotic groups are fed with non-treated feed at this time point). (**A**) Values represent the mean and SD of five samples except for group fed with phage-sprayed feed (n=4). Differences are tested by ANOVA or Kruskal-Wallis. (**B**) P-values adjusted for multiple comparison are also presented. C: control feed group; An: antibiotic feed group; PI: phage-immobilized feed group; PS: phage-sprayed feed group. Dpi: days post infection.

% mean abundance at phylum level

Feed group	Dpi	Firmicutes		Proteobacteria		Actinobacteria		Bacteroidetes		Cyanobacteria	
		Mean (%)	SD	Mean (%)	SD	Mean (%)	SD	Mean (%)	SD	Mean (%)	SD
**C**	**-1**	**52.4**	11.4	**23.0**	16.0	**16.0**	12.0	**2.4**	2.6	**1.3**	1.4
**An**	**-1**	**58.7**	17.3	**19.0**	13.0	**16.0**	2.8	**2.2**	2.8	**1.8**	1.5
**PI**	**-1**	**53.1**	31.3	**33.0**	24.1	**8.4**	3.4	**3.8**	4.9	**0.2**	0.1
**PS**	**-1**	**27.4**	31.5	**51.6**	25.7	**11.3**	4.2	**4.6**	3.2	**1.1**	1.1

A

P-values adjusted for multiple comparison

	Firmicutes	Proteobacteria	Actinobacteria	Bacteroidetes	Cyanobacteria
**C vs An**	>0.9999	0.9798	0.5886	>0.9999	0.8593
**C vs PI**	>0.9999	0.7755	0.5323	>0.9999	0.3400
**C vs PS**	0.7807	0.1240	>0.9999	0.9810	0.9913

B

Supplementary Table 3. Top seven most abundant classes at day -1 in the feed groups (note that fish in the control and antibiotic groups are fed with non-treated feed at this time point). (**A**) Values represent the mean and SD of five samples except for group fed with phage-sprayed feed (n=4). Differences are tested by ANOVA or Kruskal-Wallis. (**B**) P-values adjusted for multiple comparison are also presented. C: control feed group; An: antibiotic feed group; PI: phage-immobilized feed group; PS: phage-sprayed feed group. Dpi: days post infection.

% mean abundance at class level

Feed group	Dpi	Bacilli		γ-proteobacteria		Actinobacteria		α-proteobacteria		Clostridia		Bacteroidia		Oxyphotobacteria	
		Mean (%)	SD	Mean (%)	SD	Mean (%)	SD	Mean (%)	SD	Mean (%)	SD	Mean (%)	SD	Mean (%)	SD
**C**	**-1**	**42.6**	9.6	**17.6**	13.5	**13.8**	12.1	**4.9**	2.8	**9.7**	5.6	**2.4**	2.6	**1.3**	1.4
**An**	**-1**	**47.9**	14.1	**13.6**	8.5	**13.4**	3.9	**5.2**	5.8	**10.6**	3.9	**2.2**	2.8	**1.8**	1.5
**PI**	**-1**	**46.2**	31.9	**23.8**	16.4	**7.5**	3.5	**8.9**	11.1	**6.7**	2.9	**3.8**	4.9	**0.2**	0.1
**PS**	**-1**	**22.5**	26.5	**38.3**	25.6	**10.2**	4.4	**10.9**	6.8	**4.9**	5.5	**4.6**	3.2	**1.1**	1.1

A

P-values adjusted for multiple comparison

	Bacilli	γ-proteobacteria	Actinobacteria	α-proteobacteria	Clostridia	Bacteroidia	Oxyphotobacteria
**C vs An**	0.9650	0.9643	0.857	>0.9999	0.9771	>0.9999	>0.9999
**C vs PI**	0.9881	0.8830	>0.9999	>0.9999	0.6174	>0.9999	0.7473
**C vs PS**	0.4192	0.1890	>0.9999	0.4929	0.3090	0.9810	>0.9999

B

Supplementary Table 4. Top-30 most abundant genera at day -1 in the feed groups (note that fish in the control and antibiotic groups are fed with non-treated feed at this time point). Values represent the mean and SD of five samples except for group fed with phage-sprayed feed (n=4). C: control feed group; An: antibiotic feed group; PI: phage-immobilized feed group; PS: phage-sprayed feed group. Dpi: days post infection. Light blue = Firmicutes; Blue = Proteobacteria; Pink = Actinobacteria; Yellow = Bacteroidetes; Orange = Euryarchaeota.

No.	*Genus*	C		An		PI		PS	
		Mean (%)	SD	Mean (%)	SD	Mean (%)	SD	Mean (%)	SD
1	*Pediococcus*	**9.9**	15.5	**15.9**	6.8	**27.3**	30.1	**8.4**	16.1
2	*Lactobacillus*	**13.4**	6.4	**10.3**	2.5	**8.7**	6.6	**5.3**	5.9
3	*Acinetobacter*	**5.0**	4.9	**3.7**	4.2	**7.3**	6.2	**13.2**	13.0
4	*Rhodococcus*	**10.1**	13.6	**5.5**	3.7	**2.1**	1.8	**1.2**	1.0
5	*f__Burkholderiaceae_OTU_7*	**3.8**	3.5	**2.3**	2.0	**4.4**	2.0	**7.9**	7.8
6	*Vagococcus*	**5.0**	1.5	**5.5**	2.5	**2.7**	2.0	**1.6**	1.9
7	*Thermomonas*	**2.7**	2.6	**1.7**	1.7	**3.1**	1.6	**3.5**	3.2
8	*Clostridium sensu stricto 7*	**2.1**	1.0	**2.5**	1.4	**2.2**	1.6	**1.6**	2.0
9	*Weissella*	**3.3**	1.6	**0.5**	0.3	**2.7**	1.9	**1.4**	1.6
10	*Carnobacterium*	**2.7**	1.3	**3.0**	1.6	**1.0**	0.8	**1.1**	0.9
11	*Gordonia*	**1.3**	1.1	**1.6**	1.8	**2.3**	1.2	**1.9**	2.2
12	*Streptococcus*	**2.5**	1.6	**1.5**	1.0	**1.0**	1.3	**2.0**	2.1
13	*f__Weeksellaceae_OTU_25*	**1.3**	1.2	**1.7**	2.7	**1.8**	1.7	**1.7**	2.6
14	*Paracoccus*	**1.0**	1.5	**1.2**	1.5	**1.1**	1.3	**3.1**	2.9
15	*Photobacterium*	**1.8**	2.1	**2.5**	2.6	**0.5**	0.4	**0.6**	0.5
16	*Enhydrobacter*	**0.6**	0.6	**1.1**	0.8	**0.8**	0.7	**2.5**	2.1
17	*Pseudorhodobacter*	**0.0**	0.1	**0.3**	0.5	**3.9**	8.6	**0.2**	0.6
18	*f__Mitochondria_OTU_12*	**1.6**	2.6	**1.9**	2.4	**0.4**	0.5	**0.2**	0.1
19	*Corynebacterium 1*	**0.5**	0.4	**1.2**	0.3	**1.3**	2.0	**1.8**	1.8
20	*Stenotrophomonas*	**0.0**	0.1	**0.0**	0.0	**2.9**	4.5	**1.8**	1.7
21	*Clostridium sensu stricto 18*	**1.8**	1.4	**1.0**	0.6	**0.5**	0.3	**1.1**	1.4
22	*f__Coriobacteriales Incertae Sedis_OTU_23*	**1.3**	0.7	**1.6**	1.2	**0.7**	0.7	**0.5**	0.7
23	*Staphylococcus*	**0.4**	0.4	**2.7**	0.9	**0.4**	0.5	**0.5**	0.6
24	*Peptoniphilus*	**0.8**	0.7	**1.4**	0.4	**0.8**	0.4	**0.3**	0.4
25	*Methanosaeta*	**1.5**	3.4	**0.3**	0.4	**0.0**	0.0	**1.8**	3.5
26	*Sphingomonas*	**0.0**	0.1	**0.0**	0.1	**0.2**	0.3	**3.7**	6.5
27	*Tepidimicrobium*	**1.1**	1.4	**1.0**	0.8	**0.4**	0.4	**0.5**	0.7
28	*Enterococcus*	**0.9**	0.6	**1.2**	0.6	**0.4**	0.3	**0.4**	0.4
29	*Pseudomonas*	**0.4**	0.7	**0.1**	0.1	**1.2**	2.4	**1.4**	1.9
30	*Leuconostoc*	**0.9**	0.5	**0.7**	0.5	**0.4**	0.4	**0.8**	1.1

Supplementary Table 5. Top five most abundant phyla 1 dpi in the feed groups (note that fish in the control and antibiotic groups are fed with non-treated feed at this time point). (**A**) Values represent the mean and SD of five samples. Differences are tested by Kruskal-Wallis. When significant, differences are presented with different letters in red. (**B**) P-values adjusted for multiple comparison are presented. Significant P-values are in bold. C: control feed group; An: antibiotic feed group; PI: phage-immobilized feed group; PS: phage-sprayed feed group; C/Fp: control feed group + *F. psychrophilum*; An/Fp: antibiotic feed group + *F. psychrophilum*; PI/Fp: phage-immobilized feed group + *F. psychrophilum*; PS/Fp: phage-sprayed feed group + *F. psychrophilum*. Dpi: days post infection.

% mean abundance at phylum level

Feed group	Dpi	Firmicutes		Proteobacteria		Actinobacteria		Bacteroidetes		Cyanobacteria	
		Mean (%)	SD	Mean (%)	SD	Mean (%)	SD	Mean (%)	SD	Mean (%)	SD
**C**	**1**	**66.0** [a]	12.0	**22.1**	8.2	**7.0** [a]	3.9	**1.5**	0.1	**2.2** [ab]	4.5
**An**	**1**	**46.2** [ab]	24.0	**23.2**	11.1	**21.6** [b]	12.1	**3.7**	4.0	**1.0** [ab]	1.2
**PI**	**1**	**26.4** [ab]	24.5	**53.9**	27.8	**8.3** [ab]	6.0	**4.7**	3.4	**2.6** [ab]	3.5
**PS**	**1**	**49.2** [ab]	23.9	**34.1**	18.8	**8.0** [ab]	5.8	**2.9**	2.6	**2.0** [a]	1.8
**C/Fp**	**1**	**36.0** [ab]	15.3	**39.9**	15.7	**10.7** [ab]	3.1	**5.8**	2.7	**4.9** [ab]	9.8
**An/Fp**	**1**	**32.7** [ab]	18.0	**47.1**	15.1	**12.0** [ab]	5.7	**5.0**	5.3	**1.0** [ab]	1.7
**PI/Fp**	**1**	**59.1** [ab]	18.4	**24.3**	15.6	**12.0** [ab]	6.0	**1.9**	1.0	**0.6** [ab]	0.4
**PS/Fp**	**1**	**10.2** [b]	12.0	**61.8**	23.4	**19.8** [ab]	28.0	**4.1**	4.2	**0.02** [b]	0.1

A

P-values adjusted for multiple comparison

	Firmicutes	Proteobacteria	Actinobacteria	Bacteroidetes	Cyanobacteria
**C vs An**	>0.9999	>0.9999	**0.0373**	>0.9999	>0.9999
**C vs PI**	0.0712	0.3835	>0.9999	0.6925	>0.9999
**C vs PS**	>0.9999	>0.9999	>0.9999	>0.9999	>0.9999
**C vs C/Fp**	0.3172	0.9577	>0.9999	0.1615	>0.9999
**C vs An/Fp**	0.2441	0.3835	0.6547	>0.9999	>0.9999
**C vs PI/Fp**	>0.9999	>0.9999	>0.9999	>0.9999	>0.9999
**C vs Ps/Fp**	**0.0020**	0.0519	>0.9999	>0.9999	0.6309
**C/Fp vs An/Fp**	>0.9999	>0.9999	>0.9999	>0.9999	>0.9999
**C/Fp vs PI/Fp**	0.7000	0.7387	>0.9999	0.3088	>0.9999
**C/Fp vs Ps/Fp**	0.6275	>0.9999	>0.9999	>0.9999	0.2422
**An vs An/Fp**	>0.9999	0.3951	>0.9999	>0.9999	>0.9999
**PI vs PI/Fp**	0.1956	0.2898	>0.9999	>0.9999	>0.9999
**PS vs Ps/Fp**	0.0660	0.5005	>0.9999	>0.9999	**0.0346**

B

Supplementary Table 6. Top seven most abundant classes 1 dpi in the feed groups (note that fish in the control and antibiotic groups are fed with non-treated feed at this time point). (**A**) Values represent the mean and SD of five samples. Differences are tested by Kruskal-Wallis. When significant, differences are presented with different letters in red. (**B**) P-values adjusted for multiple comparison are also presented. Significant P-values are in bold. C: control feed group; An: antibiotic feed group; PI: phage-immobilized feed group; PS: phage-sprayed feed group; C/Fp: control feed group + *F. psychrophilum*; An/Fp: antibiotic feed group + *F. psychrophilum*; PI/Fp: phage-immobilized feed group + *F. psychrophilum*; PS/Fp: phage-sprayed feed group + *F. psychrophilum*. Dpi: days post infection.

% mean abundance at class level

Feed group	Dpi	Bacilli		γ-proteobacteria		Actinobacteria		α-proteobacteria		Clostridia		Bacteroidia		Oxyphotobacteria	
		Mean (%)	SD	Mean (%)	SD	Mean (%)	SD	Mean (%)	SD	Mean (%)	SD	Mean (%)	SD	Mean (%)	SD
**C**	**1**	**53.1** [a]	11.7	**15.7** [a]	5.1	**4.9**	4.1	**6.3**	5.3	**12.8** [a]	3.8	**1.5**	0.1	**2.2** [ab]	4.5
**An**	**1**	**36.4** [ab]	18.0	**17.4** [ab]	9.6	**18.6**	13.4	**5.6**	3.8	**9.5** [ab]	5.8	**3.7**	3.9	**1.0** [ab]	1.2
**PI**	**1**	**17.2** [ab]	18.4	**44.7** [ab]	26.3	**7.6**	5.2	**8.5**	5.2	**8.9** [ab]	8.0	**4.7**	3.4	**2.6** [ab]	3.5
**PS**	**1**	**36.3** [ab]	27.7	**26.3** [ab]	15.3	**5.9**	4.9	**7.8**	8.1	**12.7** [a]	5.3	**2.9**	2.6	**2.0** [a]	1.8
**C/Fp**	**1**	**23.5** [ab]	12.0	**31.7** [ab]	12.7	**8.9**	3.3	**7.4**	3.1	**12.2** [ab]	6.9	**5.8**	2.7	**4.9** [ab]	9.8
**An/Fp**	**1**	**27.1** [ab]	13.5	**34.9** [ab]	15.9	**10.7**	5.6	**11.5**	6.1	**5.4** [ab]	5.6	**5.0**	5.3	**1.0** [ab]	1.7
**PI/Fp**	**1**	**48.4** [ab]	14.6	**12.3** [ab]	14.1	**9.3**	6.8	**4.9**	4.3	**10.5** [ab]	4.6	**1.9**	1.0	**0.6** [ab]	0.4
**PS/Fp**	**1**	**8.7** [b]	9.1	**50.0** [b]	17.4	**19.2**	28.1	**11.8**	13.7	**1.3** [b]	2.7	**4.1**	4.2	**0.02** [b]	0.1

A

P-values adjusted for multiple comparison

	Bacilli	γ-proteobacteria	Actinobacteria	α-proteobacteria	Clostridia	Bacteroidia	Oxyphotobacteria
**C vs An**	>0.9999	>0.9999	0.0968	>0.9999	>0.9999	>0.9999	>0.9999
**C vs PI**	0.0519	0.1211	>0.9999	>0.9999	>0.9999	0.6925	>0.9999
**C vs PS**	>0.9999	>0.9999	>0.9999	>0.9999	>0.9999	>0.9999	>0.9999
**C vs C/Fp**	0.1991	0.4338	>0.9999	>0.9999	>0.9999	0.1615	>0.9999
**C vs An/Fp**	0.3381	0.3381	0.5839	>0.9999	0.6924	>0.9999	>0.9999
**C vs PI/Fp**	>0.9999	>0.9999	>0.9999	>0.9999	>0.9999	>0.9999	>0.9999
**C vs Ps/Fp**	**0.0056**	**0.0224**	>0.9999	>0.9999	**0.0459**	>0.9999	0.6309
**C/Fp vs An/Fp**	>0.9999	>0.9999	>0.9999	>0.9999	>0.9999	>0.9999	>0.9999
**C/Fp vs PI/Fp**	0.3287	0.5936	>0.9999	>0.9999	>0.9999	0.3088	>0.9999
**C/Fp vs Ps/Fp**	>0.9999	>0.9999	>0.9999	>0.9999	0.0862	>0.9999	0.2422
**An vs An/Fp**	>0.9999	0.3719	>0.9999	0.5611	>0.9999	>0.9999	>0.9999
**PI vs PI/Fp**	0.0964	0.1828	>0.9999	>0.9999	>0.9999	>0.9999	>0.9999
**PS vs Ps/Fp**	0.2388	0.5005	>0.9999	>0.9999	**0.0259**	>0.9999	**0.0346**

B

Supplementary Table 7. Top-30 most abundant genera 1 dpi in the feed groups (note that fish in the control and antibiotic groups are fed with non-treated feed at this time point). Values represent the mean and SD of five samples. C: control feed group; An: antibiotic feed group; PI: phage-immobilized feed group; PS: phage-sprayed feed group; C/Fp: control feed group + *F. psychrophilum*; An/Fp: antibiotic feed group + *F. psychrophilum*; PI/Fp: phage-immobilized feed group + *F. psychrophilum*; PS/Fp: phage-sprayed feed group + *F. psychrophilum*. Dpi: days post infection. Light blue = Firmicutes; Blue = Proteobacteria; Pink = Actinobacteria; Yellow = Bacteroidetes; Green = Cyanobacteria.

No.	*Genus*	C		An		PI		PS		C/Fp		An/Fp		PI/Fp		PS/Fp	
		Mean (%)	SD	Mean (%)	SD	Mean (%)	SD	Mean (%)	SD	Mean (%)	SD	Mean (%)	SD	Mean (%)	SD	Mean (%)	SD
1	*Lactobacillus*	**22.7**	5.8	**10.6**	4.5	**5.7**	6.2	**8.3**	4.6	**8.4**	5.5	**4.0**	3.5	**11.1**	4.2	**2.1**	4.1
2	*Pediococcus*	**7.4**	5.5	**10.1**	5.7	**1.6**	2.3	**14.9**	20.1	**1.3**	1.4	**12.2**	9.9	**14.8**	8.7	**0.0**	0.1
3	*Acinetobacter*	**3.7**	1.2	**3.0**	2.0	**7.6**	5.3	**3.4**	1.2	**7.4**	4.4	**7.8**	7.5	**3.7**	3.4	**14.0**	5.0
4	*f__Burkholderiaceae_OTU_7*	**2.8**	2.0	**2.2**	1.6	**5.7**	6.4	**3.9**	1.1	**8.7**	5.4	**8.5**	6.3	**4.5**	1.4	**7.0**	5.5
5	*Stenotrophomonas*	**1.5**	1.5	**1.1**	0.7	**11.4**	10.8	**3.4**	3.2	**0.6**	0.7	**1.9**	1.5	**2.1**	1.2	**6.6**	6.9
6	*Thermomonas*	**1.7**	0.8	**2.8**	2.1	**3.7**	2.0	**1.9**	1.2	**5.8**	2.5	**4.2**	1.7	**1.6**	1.0	**6.0**	4.2
7	*Vagococcus*	**7.2**	2.9	**5.4**	2.5	**1.8**	2.6	**3.0**	1.9	**3.3**	1.4	**2.3**	2.9	**4.3**	1.9	**0.6**	1.3
8	*Rhodococcus*	**2.0**	3.2	**9.5**	10.3	**3.5**	6.4	**2.5**	3.6	**0.5**	0.2	**2.3**	3.5	**5.0**	5.5	**1.0**	2.2
9	*Gordonia*	**0.6**	0.6	**3.1**	2.1	**2.4**	1.9	**1.0**	1.0	**4.2**	2.5	**2.8**	2.2	**1.4**	1.1	**3.9**	3.1
10	*Clostridium sensu stricto 7*	**3.6**	2.2	**2.1**	1.3	**1.4**	1.9	**4.2**	3.2	**1.9**	0.9	**1.8**	2.3	**3.3**	1.5	**0.4**	0.9
11	*Weissella*	**1.4**	0.6	**0.8**	0.8	**1.5**	2.1	**2.1**	1.6	**2.7**	1.7	**0.6**	0.4	**5.7**	2.1	**1.2**	1.6
12	*Paracoccus*	**0.5**	0.3	**2.0**	3.7	**1.9**	2.6	**0.6**	0.4	**2.6**	1.8	**2.4**	2.1	**2.3**	3.3	**5.1**	10.9
13	*Photobacterium*	**1.9**	2.3	**2.8**	2.4	**2.9**	4.3	**5.7**	4.5	**2.1**	3.2	**0.9**	1.3	**0.5**	0.7	**0.1**	0.2
14	*Rothia*	**0.0**	0.0	**0.0**	0.1	**0.0**	0.0	**0.0**	0.0	**0.0**	0.0	**0.1**	0.2	**0.5**	1.2	**12.6**	28.2
15	*Delftia*	**0.6**	0.4	**1.5**	2.4	**2.3**	3.2	**2.8**	3.2	**0.4**	0.6	**1.9**	1.8	**1.4**	1.8	**2.6**	3.5
16	*Streptococcus*	**2.7**	0.6	**0.9**	0.6	**1.6**	2.0	**2.0**	1.2	**1.7**	1.1	**0.9**	1.1	**2.4**	0.9	**1.0**	1.2
17	*f__Weeksellaceae_OTU_25*	**1.0**	0.8	**1.4**	1.3	**2.4**	2.1	**1.0**	0.8	**3.5**	1.9	**2.2**	2.2	**0.6**	0.5	**0.6**	0.4
18	*Carnobacterium*	**2.8**	1.3	**2.0**	1.4	**1.8**	1.4	**1.8**	1.6	**1.2**	1.0	**0.8**	0.6	**1.8**	1.1	**0.5**	1.1
19	*Staphylococcus*	**0.5**	0.2	**1.6**	1.4	**0.5**	0.5	**0.5**	0.9	**1.2**	0.9	**4.4**	6.6	**1.8**	1.0	**1.1**	1.6
20	*Clostridium sensu stricto 18*	**2.5**	1.1	**1.3**	1.2	**0.8**	1.2	**2.1**	1.3	**1.2**	0.9	**0.3**	0.6	**1.9**	1.7	**1.2**	0.4
21	*Pseudomonas*	**1.4**	1.1	**0.4**	0.1	**3.0**	2.8	**0.9**	0.9	**0.6**	0.5	**0.7**	0.5	**0.4**	0.3	**2.1**	2.0
22	*f__Coriobacteriales Incertae Sedis_OTU_23*	**1.1**	0.6	**2.4**	1.7	**0.5**	0.7	**1.2**	1.5	**1.3**	0.5	**0.6**	1.1	**2.2**	1.7	**0.2**	0.5
23	*Enhydrobacter*	**0.2**	0.4	**0.4**	0.4	**0.2**	0.3	**0.9**	1.3	**1.4**	0.9	**2.3**	1.5	**0.4**	0.4	**2.2**	2.3
24	*Diaphorobacter*	**0.6**	0.3	**1.1**	1.4	**0.5**	0.4	**0.7**	1.1	**1.1**	0.6	**0.6**	0.7	**0.3**	0.4	**3.1**	2.4
25	*o__Chloroplast_OTU_35*	**0.5**	0.8	**0.5**	0.5	**0.9**	1.4	**0.9**	1.0	**4.3**	9.0	**0.2**	0.1	**0.4**	0.5	**0.0**	0.1
26	*Tepidimicrobium*	**1.5**	0.6	**0.9**	1.1	**1.0**	1.5	**1.0**	0.8	**1.2**	0.8	**0.4**	0.9	**1.5**	1.0	**0.2**	0.5
27	*f__Mitochondria_OTU_12*	**0.6**	1.0	**2.0**	3.4	**2.2**	3.3	**1.0**	1.2	**0.6**	1.6	**0.4**	0.8	**0.4**	0.4	**0.0**	0.0
28	*Pseudorhodobacter*	**3.1**	4.2	**0.2**	0.3	**0.4**	0.7	**2.9**	6.5	**0.2**	0.4	**0.0**	0.0	**0.2**	0.5	**0.0**	0.0
29	*o__Chloroplast_OTU_27*	**1.7**	3.8	**0.5**	0.9	**1.7**	2.5	**1.1**	1.2	**0.5**	0.7	**0.8**	1.7	**0.2**	0.1	**0.0**	0.0
30	*Peptoniphilus*	**1.2**	0.7	**1.1**	1.0	**0.3**	0.5	**0.9**	1.4	**1.5**	1.1	**0.4**	0.4	**0.5**	0.4	**0.1**	0.3

Supplementary Table 8. Top five most abundant phyla 8 dpi in the control and antibiotic feed groups. (**A**) Values represent the mean and SD of five samples. Differences are tested by ANOVA or Kruskal-Wallis. When significant, differences are presented with different letters in red. (**B**) P-values adjusted for multiple comparison are presented. Significant P-values are in bold. C: control feed group; An: antibiotic feed group; C/Fp: control feed group + *F. psychrophilum*; An/Fp: antibiotic feed group + *F. psychrophilum*. Dpi: days post infection.

% mean abundance at phylum level

Feed group	Dpi	Firmicutes		Proteobacteria		Actinobacteria		Bacteroidetes		Cyanobacteria	
		Mean (%)	SD	Mean (%)	SD	Mean (%)	SD	Mean (%)	SD	Mean (%)	SD
**C**	**8**	**40.8**	15.8	**22.7**	9.6	**33.1** [a]	7.9	**0.5** [a]	0.6	**0.7** [ab]	0.6
**An**	**8**	**31.0**	9.9	**24.6**	17.9	**39.4** [a]	26.0	**1.5** [ac]	2.1	**2.3** [a]	1.4
**C/Fp**	**8**	**21.1**	18.3	**34.0**	21.7	**9.2** [b]	7.7	**32.6** [b]	39.3	**0.1** [b]	0.2
**An/Fp**	**8**	**31.4**	25.1	**45.8**	17.7	**8.6** [b]	1.5	**9.1** [bc]	6.8	**2.7** [a]	2.0

A

P-values adjusted for multiple comparison

	Firmicutes	Proteobacteria	Actinobacteria	Bacteroidetes	Cyanobacteria
**C vs An**	0.7271	0.9961	0.8223	>0.9999	0.6463
**C vs C/Fp**	0.2391	0.6102	**0.0425**	**0.0083**	0.5345
**C vs An/Fp**	0.7478	0.1239	**0.0375**	**0.036**	0.5884
**An vs C/Fp**	0.7257	0.7257	**0.0102**	**0.0485**	**0.0292**
**An vs An/Fp**	>0.9999	0.1683	**0.0089**	0.1630	>0.9999
**C/Fp vs An/Fp**	0.7048	0.5874	0.9999	>0.9999	**0.0249**

B

Supplementary Table 9. Top seven most abundant classes 8 dpi in the control and antibiotic feed groups. (**A**) Values represent the mean and SD of five samples. Differences are tested by ANOVA or Kruskal-Wallis. When significant, differences are presented with different letters in red. (**B**) P-values adjusted for multiple comparison are presented. Significant P-values are in bold. C: control feed group; An: antibiotic feed group; C/Fp: control feed group + *F. psychrophilum*; An/Fp: antibiotic feed group + *F. psychrophilum*. Dpi: days post infection.

% mean abundance at class level

Feed group	Dpi	Bacilli		γ-proteobacteria		Actinobacteria		α-proteobacteria		Clostridia		Bacteroidia		Oxyphotobacteria	
		Mean (%)	SD	Mean (%)	SD	Mean (%)	SD	Mean (%)	SD	Mean (%)	SD	Mean (%)	SD	Mean (%)	SD
**C**	**8**	**30.7**	12.7	**9.5** a	5.2	**31.1** ab	9.3	**12.9**	10.6	**10.5** a	3.2	**0.5** a	0.6	**0.7** ab	0.6
**An**	**8**	**29.5**	9.5	**12.7** ab	12.5	**39.3** a	26.0	**11.8**	12.1	**1.5** b	0.4	**1.5** ac	2.1	**2.3** a	1.4
**C/Fp**	**8**	**15.6**	14.0	**27.3** ab	17.5	**8.1** b	6.5	**6.1**	4.0	**5.4** ab	4.7	**32.6** b	39.3	**0.1** b	0.2
**An/Fp**	**8**	**30.2**	24.0	**34.4** b	15.9	**8.4** b	1.4	**10.8**	4.7	**1.1** b	1.0	**9.1** bc	6.8	**2.7** a	2.0

A

P-values adjusted for multiple comparison

	Bacilli	γ-proteobacteria	Actinobacteria	α-proteobacteria	Clostridia	Bacteroidia	Oxyphotobacteria
**C vs An**	0.9987	0.9658	0.6889	0.9948	**0.0007**	>0.9999	0.6463
**C vs C/Fp**	0.3413	0.1407	0.0536	0.4718	0.0529	**0.0083**	0.5345
**C vs An/Fp**	>0.9999	**0.0274**	0.0562	0.9650	**0.0005**	**0.0360**	0.5884
**An vs C/Fp**	0.4052	0.2618	**0.0084**	0.5942	0.1213	**0.0485**	**0.0292**
**An vs An/Fp**	0.9997	0.0569	**0.0089**	0.9956	0.9950	0.1630	>0.9999
**C/Fp vs An/Fp**	0.3673	0.7264	>0.9999	0.7149	0.0858	>0.9999	**0.0249**

B

Supplementary Table 10. Top-30 most genera phyla 8 dpi in the control and antibiotic feed groups. Values represent the mean and SD of five samples. C: control feed group; An: antibiotic feed group; C/Fp: control feed group + *F. psychrophilum*; An/Fp: antibiotic feed group + *F. psychrophilum*. Dpi: days post infection. Light blue = Firmicutes; Blue = Proteobacteria; Pink = Actinobacteria; Yellow = Bacteroidetes; Green = Cyanobacteria.

No.	*Genus*	C		An		C/Fp		An/Fp	
		Mean (%)	SD	Mean (%)	SD	Mean (%)	SD	Mean (%)	SD
1	***Rhodococcus***	**28.5**	9.5	**37.4**	26.7	**3.6**	4.4	**2.1**	1.7
2	***Lactobacillus***	**12.0**	4.6	**15.4**	4.7	**6.4**	6.3	**16.2**	11.8
3	***Flavobacterium***	**0.0**	0.0	**0.0**	0.0	**28.5**	41.6	**0.0**	0.0
4	***f__Burkholderiaceae_OTU_7***	**2.0**	2.0	**2.1**	1.9	**6.6**	5.8	**7.8**	4.8
5	***Streptococcus***	**1.5**	0.3	**7.1**	2.9	**1.0**	1.6	**8.8**	7.9
6	***Acinetobacter***	**2.6**	2.1	**3.0**	2.9	**4.9**	3.3	**7.4**	3.5
7	***Pseudorhodobacter***	**7.8**	7.7	**8.4**	10.7	**0.7**	1.6	**0.3**	0.5
8	***Thermomonas***	**1.0**	0.7	**2.1**	2.3	**4.0**	2.6	**4.5**	3.0
9	***Weissella***	**2.7**	1.2	**3.1**	1.9	**1.5**	1.5	**2.5**	2.5
10	***Stenotrophomonas***	**0.7**	0.4	**1.0**	1.4	**2.6**	1.7	**4.1**	4.3
11	***Gordonia***	**0.8**	0.9	**0.8**	0.7	**2.1**	1.4	**4.5**	0.5
12	***Vagococcus***	**3.8**	1.4	**0.4**	0.0	**3.0**	2.5	**0.4**	0.5
13	***f__Weeksellaceae_OTU_25***	**0.3**	0.3	**0.4**	0.3	**2.8**	3.5	**3.8**	2.0
14	***o__Chloroplast_OTU_35***	**0.5**	0.4	**2.0**	1.3	**0.1**	0.3	**2.6**	2.0
15	***Pediococcus***	**4.1**	4.5	**0.1**	0.2	**0.9**	0.9	**0.0**	0.0
16	***Allorhizobium-Neorhizobium-Pararhizobium-Rhizobium***	**1.2**	1.8	**0.9**	0.7	**0.4**	0.6	**2.5**	2.3
17	***Paracoccus***	**0.3**	0.4	**0.8**	1.0	**1.7**	0.8	**1.7**	0.8
18	***Clostridium sensu stricto 7***	**2.8**	0.7	**0.1**	0.2	**1.4**	2.1	**0.1**	0.1
19	***Delftia***	**0.3**	0.4	**0.3**	0.4	**1.0**	0.7	**2.5**	3.0
20	***Pseudomonas***	**0.5**	0.4	**1.0**	1.9	**1.5**	1.9	**1.1**	2.2
21	***Clostridium sensu stricto 18***	**2.1**	1.4	**0.0**	0.1	**1.4**	1.5	**0.0**	0.1
22	***Carnobacterium***	**1.9**	0.8	**0.3**	0.3	**1.1**	1.0	**0.1**	0.2
23	***Bosea***	**1.6**	1.4	**0.3**	0.6	**1.6**	0.8	**0.7**	0.4
24	***Diaphorobacter***	**0.3**	0.4	**0.4**	0.8	**1.1**	0.7	**0.9**	1.1
25	***f__Rhizobiaceae_OTU_81***	**0.2**	0.3	**0.0**	0.1	**0.8**	1.2	**1.5**	1.3
26	***Enhydrobacter***	**0.2**	0.2	**0.1**	0.2	**1.4**	1.3	**0.8**	0.7
27	***Bacillus***	**0.6**	0.3	**0.8**	0.7	**0.4**	0.6	**0.7**	0.7
28	***f__Xanthomonadaceae_OTU_65***	**0.1**	0.2	**0.9**	1.8	**1.3**	0.4	**1.1**	1.7
29	***Peptoniphilus***	**1.1**	0.8	**0.0**	0.1	**1.0**	0.9	**0.2**	0.3
30	***Leadbetterella***	**0.1**	0.1	**0.7**	1.6	**0.0**	0.1	**1.4**	3.2

Supplementary Table 11. Top five most abundant phyla 33 dpi in the feed groups (note that fish in the control and antibiotic groups are fed with non-treated feed at this time point). (**A**) Values represent the mean and SD of five samples. Differences are tested by Kruskal-Wallis. When significant, differences are presented with different letters in red. (**B**) P-values adjusted for multiple comparison are presented. Significant P-values are in bold. C: control feed group; An: antibiotic feed group; PI: phage-immobilized feed group; PS: phage-sprayed feed group; C/Fp: control feed group + *F. psychrophilum*; An/Fp: antibiotic feed group + *F. psychrophilum*; PI/Fp: phage-immobilized feed group + *F. psychrophilum*; PS/Fp: phage-sprayed feed group + *F. psychrophilum*. Dpi: days post infection.

% mean abundance at phylum level

Feed group	Dpi	Firmicutes		Proteobacteria		Actinobacteria		Bacteroidetes		Cyanobacteria	
		Mean (%)	SD	Mean (%)	SD	Mean (%)	SD	Mean (%)	SD	Mean (%)	SD
**C**	**33**	**32.1**	23.3	**37.9**	13.3	**23.4**	9.5	**0.9**	0.5	**2.9** ^{ab}	2.4
**An**	**33**	**29.9**	21.5	**40.8**	16.8	**23.3**	13.6	**0.5**	0.3	**2.0** ^{ab}	1.6
**PI**	**33**	**37.3**	19.7	**30.3**	15.3	**26.4**	11.9	**0.8**	0.4	**1.6** ^{ab}	1.9
**PS**	**33**	**65.0**	10.9	**18.8**	9.4	**10.5**	2.7	**1.0**	1.0	**3.1** ^{ab}	1.4
**C/Fp**	**33**	**25.9**	25.7	**39.1**	19.1	**25.1**	12.8	**3.8**	5.6	**0.5** ^{a}	0.5
**An/Fp**	**33**	**10.7**	8.1	**54.5**	10.8	**22.6**	7.2	**1.3**	1.2	**4.8** ^{ab}	5.6
**PI/Fp**	**33**	**36.9**	29.8	**41.4**	20.2	**15.8**	6.4	**0.5**	0.4	**0.6** ^{ab}	1.4
**PS/Fp**	**33**	**49.0**	2.7	**31.8**	7.5	**9.8**	9.0	**0.8**	0.7	**6.4** ^{b}	3.0

A

P-values adjusted for multiple comparison

	Firmicutes	Proteobacteria	Actinobacteria	Bacteroidetes	Cyanobacteria
**C vs An**	>0.9999	>0.9999	>0.9999	>0.9999	>0.9999
**C vs PI**	>0.9999	>0.9999	>0.9999	>0.9999	>0.9999
**C vs PS**	0.3602	0.2974	0.2282	>0.9999	>0.9999
**C vs C/Fp**	>0.9999	>0.9999	>0.9999	>0.9999	0.8613
**C vs An/Fp**	0.8167	>0.9999	>0.9999	>0.9999	>0.9999
**C vs PI/Fp**	>0.9999	>0.9999	>0.9999	>0.9999	0.7317
**C vs Ps/Fp**	>0.9999	>0.9999	0.1211	>0.9999	0.7317
**C/Fp vs An/Fp**	>0.9999	0.9100	>0.9999	>0.9999	0.3716
**C/Fp vs PI/Fp**	>0.9999	>0.9999	>0.9999	0.6271	>0.9999
**C/Fp vs Ps/Fp**	>0.9999	>0.9999	0.0611	>0.9999	**0.0093**
**An vs An/Fp**	>0.9999	>0.9999	>0.9999	>0.9999	>0.9999
**PI vs PI/Fp**	>0.9999	>0.9999	>0.9999	>0.9999	>0.9999
**PS vs Ps/Fp**	0.9100	>0.9999	>0.9999	>0.9999	>0.9999

B

Supplementary Table 12. Top seven most abundant classes 33 dpi in the feed groups (note that fish in the control and antibiotic groups are fed with non-treated feed at this time point). (**A**) Values represent the mean and SD of five samples. Differences are tested by Kruskal-Wallis. When significant, differences are presented with different letters in red. (**B**) P-values adjusted for multiple comparison are also presented. Significant P-values are in bold. C: control feed group; An: antibiotic feed group; PI: phage-immobilized feed group; PS: phage-sprayed feed group; C/Fp: control feed group + *F. psychrophilum*; An/Fp: antibiotic feed group + *F. psychrophilum*; PI/Fp: phage-immobilized feed group + *F. psychrophilum*; PS/Fp: phage-sprayed feed group + *F. psychrophilum*. Dpi: days post infection.

% mean abundance at class level

Feed group	Dpi	Bacilli		γ-proteobacteria		Actinobacteria		α-proteobacteria		Clostridia		Bacteroidia		Oxyphotobacteria	
		Mean (%)	SD	Mean (%)	SD	Mean (%)	SD	Mean (%)	SD	Mean (%)	SD	Mean (%)	SD	Mean (%)	SD
**C**	**33**	**24.0**	19.6	**17.6**	7.2	**22.4**	10.2	**20.2**	17.7	**8.1**	7.2	**0.9**	0.5	**2.9** ab	2.4
**An**	**33**	**22.2**	16.3	**10.5**	4.0	**21.7**	14.2	**30.2**	17.7	**7.5**	5.5	**0.5**	0.3	**2.0** ab	1.6
**PI**	**33**	**28.8**	14.4	**13.4**	9.1	**24.5**	12.2	**16.9**	17.1	**8.4**	6.0	**0.8**	0.4	**1.6** ab	1.9
**PS**	**33**	**48.9**	7.2	**11.9**	5.8	**7.4**	2.9	**6.9**	4.8	**15.9**	3.8	**1.0**	1.0	**3.1** ab	1.4
**C/Fp**	**33**	**20.0**	19.7	**17.0**	17.3	**23.7**	13.5	**22.0**	24.5	**5.7**	5.9	**3.8**	5.6	**0.5** a	0.5
**An/Fp**	**33**	**6.8**	5.4	**12.1**	10.2	**21.7**	7.1	**42.6**	19.3	**3.8**	2.9	**1.3**	1.2	**4.8** ab	5.6
**PI/Fp**	**33**	**26.6**	21.4	**19.6**	11.3	**23.6**	6.1	**22.7**	23.8	**10.7**	9.0	**0.5**	0.4	**0.6** ab	1.4
**PS/Fp**	**33**	**32.8**	3.9	**19.5**	5.5	**8.6**	8.3	**11.9**	6.3	**16.1**	2.5	**0.8**	0.7	**6.4** b	3.0

A

P-values adjusted for multiple comparison

	Bacilli	γ-proteobacteria	Actinobacteria	α-proteobacteria	Clostridia	Bacteroidia	Oxyphotobacteria
**C vs An**	>0.9999	>0.9999	>0.9999	>0.9999	>0.9999	>0.9999	>0.9999
**C vs PI**	>0.9999	>0.9999	>0.9999	>0.9999	>0.9999	>0.9999	>0.9999
**C vs PS**	0.2282	>0.9999	0.1615	>0.9999	0.5839	>0.9999	>0.9999
**C vs C/Fp**	>0.9999	>0.9999	>0.9999	>0.9999	>0.9999	>0.9999	0.8613
**C vs An/Fp**	0.8167	>0.9999	>0.9999	0.7321	>0.9999	>0.9999	>0.9999
**C vs PI/Fp**	>0.9999	>0.9999	>0.9999	>0.9999	>0.9999	>0.9999	0.7317
**C vs Ps/Fp**	>0.9999	>0.9999	0.1302	>0.9999	0.408	>0.9999	0.7317
**C/Fp vs An/Fp**	>0.9999	>0.9999	>0.9999	0.4453	>0.9999	>0.9999	0.3716
**C/Fp vs PI/Fp**	>0.9999	>0.9999	>0.9999	>0.9999	>0.9999	0.6271	>0.9999
**C/Fp vs Ps/Fp**	>0.9999	>0.9999	0.0769	>0.9999	0.0611	>0.9999	**0.0093**
**An vs An/Fp**	>0.9999	>0.9999	>0.9999	>0.9999	>0.9999	>0.9999	>0.9999
**PI vs PI/Fp**	>0.9999	>0.9999	>0.9999	>0.9999	>0.9999	>0.9999	>0.9999
**PS vs Ps/Fp**	0.5301	0.663	>0.9999	>0.9999	>0.9999	>0.9999	>0.9999

B

Supplementary Table 13. Top-30 most abundant genera 33 dpi in the feed groups (note that fish in the control and antibiotic groups are fed with non-treated feed at this time point). Values represent the mean and SD of five samples. C: control feed group; An: antibiotic feed group; PI: phage-immobilized feed group; PS: phage-sprayed feed group; C/Fp: control feed group + *F. psychrophilum*; An/Fp: antibiotic feed group + *F. psychrophilum*; PI/Fp: phage-immobilized feed group + *F. psychrophilum*; PS/Fp: phage-sprayed feed group + *F. psychrophilum*. Dpi: days post infection. Light blue = Firmicutes; Blue = Proteobacteria; Pink = Actinobacteria; Green = Cyanobacteria; Purple = Patescibacteria.

No.	*Genus*	C		AN		PI		PS		C/Fp		AN/Fp		PI/Fp		PS/Fp	
		Mean (%)	SD	Mean (%)	SD	Mean (%)	SD	Mean (%)	SD	Mean (%)	SD	Mean (%)	SD	Mean (%)	SD	Mean (%)	SD
1	*Rhodococcus*	**16.8**	13.1	**18.4**	13.6	**17.2**	10.4	**2.3**	1.1	**16.0**	13.4	**18.0**	7.1	**8.6**	7.1	**6.3**	7.5
2	*Lactobacillus*	**12.2**	15.7	**3.5**	4.5	**9.1**	5.8	**15.6**	6.0	**7.6**	7.2	**1.3**	1.2	**9.8**	8.1	**10.7**	1.8
3	*Pseudorhodobacter*	**0.7**	1.3	**1.7**	1.8	**11.0**	15.1	**0.0**	0.0	**14.7**	23.7	**17.7**	22.4	**10.7**	12.1	**0.0**	0.1
4	*Photobacterium*	**3.1**	3.0	**3.6**	1.9	**1.4**	1.8	**3.7**	1.8	**1.0**	1.0	**6.0**	8.1	**0.8**	1.1	**10.5**	6.3
5	*Pediococcus*	**0.1**	0.1	**7.6**	6.6	**3.5**	4.9	**10.0**	9.7	**0.9**	0.7	**1.7**	1.6	**2.1**	2.7	**3.1**	3.4
6	*f__Mitochondria_ OTU_12*	**2.2**	2.6	**1.2**	1.1	**1.0**	0.8	**1.7**	0.9	**0.7**	0.9	**10.3**	14.1	**0.6**	0.8	**7.9**	7.2
7	*Bosea*	**4.5**	4.8	**9.3**	8.7	**1.3**	1.6	**0.4**	0.3	**0.4**	0.5	**1.5**	0.7	**5.1**	7.2	**0.1**	0.1
8	*Vagococcus*	**1.4**	1.5	**2.3**	2.3	**3.6**	1.9	**5.0**	2.0	**2.7**	3.0	**0.9**	0.6	**2.6**	2.5	**2.8**	1.7
9	*Weissella*	**1.2**	1.6	**0.5**	0.6	**3.5**	1.9	**5.0**	1.4	**2.0**	2.1	**0.1**	0.1	**2.6**	3.2	**4.6**	2.3
10	*Phreatobacter*	**8.8**	11.5	**7.0**	6.1	**0.6**	0.7	**1.1**	1.3	**0.3**	0.3	**0.2**	0.1	**1.2**	1.9	**0.0**	0.0
11	*Acinetobacter*	**1.8**	1.7	**1.5**	1.4	**3.5**	3.1	**1.7**	1.7	**5.1**	6.3	**1.5**	1.5	**1.7**	1.1	**2.4**	1.5
12	*Clostridium sensu stricto 7*	**2.3**	1.5	**1.7**	1.8	**1.6**	1.5	**3.4**	1.3	**1.4**	1.4	**0.7**	0.6	**3.1**	3.5	**4.9**	1.7
13	*Streptococcus*	**2.4**	2.3	**1.2**	0.7	**2.1**	1.0	**2.7**	1.2	**2.2**	2.3	**0.8**	0.8	**2.9**	2.9	**4.3**	2.0
14	*Allorhizobium-Neorhizobium-Pararhizobium-Rhizobium*	**0.9**	1.0	**8.6**	6.9	**0.3**	0.6	**0.4**	0.6	**0.5**	0.7	**6.2**	3.6	**0.2**	0.3	**0.9**	0.5
15	*f__Burkholderiaceae_ OTU_7*	**1.7**	1.8	**1.1**	1.1	**2.0**	1.7	**1.1**	1.4	**3.3**	3.9	**1.5**	1.7	**1.8**	1.6	**2.2**	1.8
16	*Carnobacterium*	**0.8**	0.8	**1.5**	1.2	**2.3**	1.5	**3.4**	2.0	**1.4**	1.8	**0.4**	0.3	**1.5**	1.3	**0.9**	0.9
17	*Tepidimicrobium*	**1.3**	2.0	**1.0**	0.8	**1.5**	1.3	**2.7**	0.3	**0.8**	1.0	**0.4**	0.4	**1.6**	1.5	**2.7**	2.5
18	*f__Microbacteriaceae_ OTU_47*	**1.1**	1.0	**1.4**	1.7	**3.9**	2.6	**0.6**	0.9	**2.6**	4.1	**1.7**	1.1	**0.5**	0.7	**0.4**	0.4
19	*Thermomonas*	**2.9**	4.3	**0.5**	0.5	**1.9**	1.7	**0.5**	0.6	**1.8**	1.6	**1.4**	1.5	**1.4**	1.3	**1.0**	1.0
20	*o__Chloroplast_ OTU_27*	**1.3**	0.8	**0.6**	0.3	**0.5**	0.7	**1.1**	0.6	**0.3**	0.3	**3.5**	5.2	**0.0**	0.0	**4.1**	2.1
21	*o__Chloroplast_ OTU_35*	**1.5**	1.5	**1.4**	1.6	**1.1**	1.6	**2.0**	0.9	**0.2**	0.2	**1.1**	1.0	**0.1**	0.1	**2.1**	1.8
22	*Clostridium sensu stricto 18*	**0.8**	1.0	**0.6**	0.4	**1.2**	1.0	**2.7**	0.9	**0.9**	1.1	**0.4**	0.4	**0.9**	1.3	**1.7**	0.8
23	*f__Coriobacteriales Incertae Sedis_OTU_23*	**0.6**	0.7	**1.1**	1.4	**0.9**	0.6	**2.0**	1.2	**0.9**	1.1	**0.6**	0.7	**1.1**	1.0	**0.7**	0.7
24	*Stenotrophomonas*	**1.5**	2.6	**0.3**	0.2	**0.9**	1.0	**0.9**	1.0	**1.2**	1.2	**0.1**	0.1	**2.4**	2.7	**0.3**	0.1
25	*Enhydrobacter*	**0.3**	0.4	**1.1**	0.1	**0.2**	0.2	**0.2**	0.3	**0.4**	0.3	**0.1**	0.1	**6.0**	12.7	**0.5**	0.6
26	*Paracoccus*	**0.4**	0.6	**0.1**	0.1	**0.9**	1.0	**0.3**	0.6	**2.2**	3.7	**0.3**	0.3	**1.6**	1.9	**0.2**	0.2
27	*Bacillus*	**0.5**	0.8	**0.4**	0.5	**0.6**	0.7	**1.8**	0.6	**0.5**	0.6	**0.1**	0.1	**0.3**	0.3	**1.9**	1.3
28	*f__Saccharimonadaceae_ OTU_50*	**0.8**	0.7	**1.2**	0.9	**1.0**	0.4	**0.1**	0.2	**0.8**	0.3	**1.2**	1.0	**0.7**	0.8	**0.2**	0.2
29	*Lactococcus*	**2.1**	3.4	**0.4**	0.4	**0.7**	0.5	**0.9**	0.4	**0.4**	0.4	**0.2**	0.2	**0.7**	0.9	**0.4**	0.2
30	*Pseudomonas*	**0.4**	0.5	**1.3**	3.5	**0.1**	0.1	**0.4**	0.6	**1.3**	2.3	**0.1**	0.1	**0.8**	1.1	**0.3**	0.4
